# Inosine enhances the efficacy of immune‐checkpoint inhibitors in advanced solid tumors: A randomized, controlled, Phase 2 study

**DOI:** 10.1002/cam4.70143

**Published:** 2024-09-13

**Authors:** Haiqing Zhao, Wei Zhang, Yuting Lu, Yin Dong, Zhihao He, Hongchao Zhen, Qin Li

**Affiliations:** ^1^ Department of Oncology, Beijing Friendship Hospital Capital Medical University Beijing People's Republic of China; ^2^ Internal Medicine Department People's Hospital of Shen chi County Shanxi People's Republic of China

**Keywords:** gut microbiota, immune‐checkpoint inhibitor, inosine, PD‐1/PD‐L1, tumor

## Abstract

**Background:**

This study aimed to evaluate whether inosine enhances the efficacy of immune‐checkpoint inhibitors in human malignant solid tumors.

**Methods:**

This single‐center, prospective, randomized, open‐label study was conducted, from January 2021 to December 2022, in Beijing Friendship Hospital, Capital Medical University, and participants were randomly assigned (1:1) to either the inosine (trial) or non‐inosine (control) group that received inosine (dosage: 0.2 g, three times/day) + PD‐1/PD‐L1 inhibitor or only PD‐1/PD‐L1 inhibitor ± targeted ± chemotherapy, respectively. Efficacy was assessed every 6 weeks (i.e., after every two–three treatment cycles). The primary endpoint was the objective response rate (ORR); the secondary endpoints were disease control rate, overall survival (OS), and progression‐free survival (PFS). The trial was registered at ClinicalTrials.gov (NCT05809336).

**Results:**

Among the 172 participants with advanced malignant solid tumors, 86 each were assigned to the inosine and non‐inosine groups, wherein the median PFS (95% CI) was 7.00 (5.31–8.69) and 4.40 (3.10–5.70) months, respectively (hazard ratio [HR] 0.63; 95% CI 0.44–0.90, *p* = 0.011), and the ORR was 26.7% and 15.1%, respectively (*p* = 0.061). In the inosine and non‐inosine groups, the median OS was not reached and was 29.67 (95% CI 17.40–41.94) months, respectively (HR 1.05 [95% CI 0.59–1.84], *p* = 0.874). Compared with the non‐inosine group, the median PFS and ORR of the inosine group were significantly prolonged and improved in the multiple exploratory subgroup analyses. The safety analysis showed that Grades 3 and 4 adverse reactions occurred in 25 (29%) and 31 (36%) patients in the inosine and non‐inosine groups, respectively, and tended to decrease in the inosine group compared with the non‐inosine group.

**Conclusion:**

Inosine had a tendency to enhance the efficacy of immune‐checkpoint inhibitors and reduced immunotherapy‐related adverse reactions.

## Abstract 2

Compared with the control group, the median progression‐free survival was significantly prolonged and the objective response rate tended to increase in the inosine group. Inosine has a tendency to enhance the efficacy of immune‐checkpoint inhibitors and reduce immunotherapy‐related adverse reactions in clinical applications.

## 1 BACKGROUND

Immune‐checkpoint inhibitors (ICIs), as a novel tumor therapy, have led to breakthroughs in the treatment of malignant solid tumors.^1^, ^2^, ^3^, ^4^ However, there is heterogeneity in the therapeutic response to ICIs. In clinical practice, PD‐1/PD‐L1 inhibitors are the predominant ICIs, with objective response rates (ORR) of 10%–30%,^5^, ^6^, ^7^, ^8^, ^9^, ^10^, ^11^, ^12^, ^13^, ^14^, ^15^, ^16^, ^17^, ^18^ despite primary ICI resistance in some patients.^19^ Population‐level screening for ascertaining the benefits of ICI and overcoming resistance involves significant clinical challenges. The mechanisms of PD‐1/PD‐L1 inhibitor resistance include a lack of tumor immunogenicity, which results in the inability of T cells to recognize tumor cells, resistance of tumor cells to interferons, suppression of T‐cell responses by immunosuppressive factors myeloid‐derived suppressor cells (MDSC) and regulatory T (Treg) cells in the tumor microenvironment, and lack of T‐cell function.^20^

Several studies have demonstrated that gut microbes can enhance the efficacy of ICI therapy^21^, ^22^; however, the molecular mechanism thereof remain unclear. Inosine, a metabolite of the intestinal microbiota, has a key role in enhancing the ICI therapeutic efficacy.^21^, ^22^, ^23^, ^24^, ^25^, ^26^ Inosine is a natural component of human body that can restore normal cellular metabolism under low‐energy hypoxic conditions, activate pyruvate oxidase enzymes, enhance coenzyme A activity, activate cells, and stimulate metabolism.^27^, ^28^, ^29^, ^30^, ^31^ In regard to tumor immunity, inosine increases the tumor cell immunogenicity^32^, ^33^ and significantly upregulates the activation of interferon‐γ (IFN‐γ) and tumor necrosis factor‐α (TNF‐α) signaling pathways, whereby IFN‐γ can heighten antigen presentation.^33^, ^34^ Inosine enhances tumor cell immunogenicity through the direct inhibition of UBA6 from tumor cells.^33^, ^35^ By interacting with the adenosine 2A receptor on T lymphocytes, inosine promotes T‐cell activation and proliferation.^26^, ^33^ Furthermore, inosine enhances phytohemagglutinin‐mediated immune responses, increases tumor antigen levels, and enhances T‐lymphocyte differentiation and proliferation.^33^, ^36^ Moreover, inosine inhibits MDSC‐induced immunosuppression.^33^, ^35^ Finally, inosine can be used as an alternative carbon source to support the growth and function of CD8^+^ T cells.^36^ Based on its mechanism of action, inosine overcomes primary and adaptive resistance to PD‐1 and PD‐L1 inhibitors.

Despite its clinical availability for decades, inosine has not been used for tumor immunotherapy. Considered with the preclinical evidence of its synergistic effect with ICI, the therapeutic potential of inosine deserves further evaluation. To further clarify whether inosine enhances the efficacy of immunotherapy, a prospective clinical study was conducted to compare the difference in efficacy between an inosine‐treated group (trial group) and a non‐inosine group (control group) for malignant advanced solid tumors. Inosine was used to enhance the efficacy of ICI or reverse ICI resistance and thus develop a new strategy for ICI combination therapy.

## 2 MATERIALS AND METHODS

### 2.1 Study design and participants

This single‐center, prospective, randomized, open‐label, Phase 2 study was conducted at the Department of Oncology, Beijing Friendship Hospital, Capital Medical University, Beijing, People's Republic of China, was approved by the Clinical Investigation Ethics Committee of Beijing Friendship Hospital, Capital Medical University (ethical approval number 2021‐P2‐152‐02), and was conducted in accordance with the principles evinced in the Declaration of Helsinki. All participants provided written informed consent for data collection and study participation.

#### 2.1.1 Inclusion criteria

The inclusion criteria were as follows: (1) Histopathologically confirmed solid tumors that were locally advanced, recurrent, metastatic, or unresectable. (2) Age 18–75 years (3) Eastern Cooperative Oncology Group (ECOG) score ≤2. (4) The presence of at least one lesion that could be measured or evaluated according to the Response Evaluation Criteria in Solid Tumors v1.1 (RECIST v1.1). (5) Provision of written informed consent (Appendix S3: detailed inclusion criteria).

#### 2.1.2 Exclusion criteria

The exclusion criteria were as follows: (1) indication of active bleeding or perforation. (2) Systemic antitumor therapy with herbs or immunomodulatory drugs (including thymidine, interferon, and interleukin) within 2 weeks before the first dose of study treatment. (Appendix S3: detailed exclusion criteria).

### 2.2 Study procedures and treatment

The treatment regimens for the inosine and non‐inosine groups were inosine + PD‐1/PD‐L1 inhibitor or only PD‐1/PD‐L1 inhibitor ± targeted ± chemotherapy. According to the instructions, inosine tablets are used as an adjuvant treatment for acute and chronic hepatitis at a dose of 0.2–0.6 g, three times/day. After comprehensively considering the relevant factors, we chose to combine immunotherapy and inosine (dose 0.2 g, three times/day; each inosine tablet contains 0.2 g inosine and excipients, including dextrin, sucrose, hydroxypropyl cellulose, adhesive [starch pulp or ethanol], sodium carboxymethyl starch, and magnesium stearate, manufactured by Guangdong Hengjian). The relevant medications included: (1) PD‐1 inhibitors, including karelizumab (200 mg for 3 weeks), sindilizumab injection (200 mg for 3 weeks), pavelizumab (200 mg for 2 weeks), tirilizumab (200 mg for 3 weeks), pembrolizumab (200 mg for 3 weeks), and teraplizumab (3 mg/kg for 2 weeks). (2) PD‐L1 inhibitor atalizumab (1200 mg for 3 weeks). (3) PD‐1/PD‐L1 inhibitor combination regimen, including chemotherapy, targeting, or chemotherapy + targeting every 2 or 3 weeks.

### 2.3 Assessments and follow‐up

Antitumor efficacy was evaluated, according to RECIST v1.1, every 6 weeks, using computed tomography or magnetic resonance imaging and tumor markers. The treatment was administered until disease progression, death, or unacceptable toxic side effects; the investigator concluded that there was no benefit with continued treatment; other criteria for discontinuation were met (e.g., pregnancy, individual patient reasons, or concurrent illness); and the participant chose to withdraw from the study, withdrew informed consent, or was lost to follow‐up.

### 2.4 Sample size calculation

In this study, the unilateral *β* = 0.10 was considered (Power = 0.90; Alpha = 0.05); the samples of the two groups were equal, N1 (trial group) = N2 (control group), the predicted ORR of the combined group was 40%, and the ORR of the single group was 20%, and the number of cases in each group was calculated as 89, considering a dropout rate of approximately 5%, a total sample size of 188, with 94 participants in each group, was selected. The final specimen volume figures for the inquiry process were used.

### 2.5 Outcomes

The primary endpoint was the ORR, the secondary endpoints were the disease control rate (DCR), overall survival (OS), and progression‐free survival (PFS). Efficacy assessments were jointly completed after every two–three treatment cycles (every 6 weeks) and were undertaken by two experienced attending doctors in accordance with RECIST v1.1. The ORR represents the proportion of patients with the best overall effect evaluation of CR and PR, and the DCR denotes the proportion of patients with the best overall effect evaluation of CR, PR, and SD. The OS is the time from randomization grouping to death from any cause, PFS is the time from the start of randomization to the first documented disease progression or death from any cause.

Patients who received at least one treatment cycle were evaluated for safety. Adverse events were graded according to the National Cancer Institute Common Terminology Criteria for Adverse Events (CTCAE) version 5.0. The occurrence of adverse events and immune‐related adverse events throughout the study period and for up to 30 days (90 days for serious adverse events) after treatment discontinuation was monitored. Additionally, a drug‐related assessment was performed to evaluate the safety in the inosine and non‐inosine groups.

### 2.6 Statistical analysis

Quantitative information for non‐normal distributions was described using lower quartile, median, and upper quartile statistics, and qualitative information was described using absolute numbers and constitutive ratios. The Kaplan–Meier method was used to calculate the OS and PFS of the inosine and the non‐inosine groups, and the log‐rank test was used to ascertain the intergroup differences in OS and PFS. The chi‐squared test was used to compare the differences in ORR, DCR, PFS, and OS rates for both groups. Receiver operating characteristic curves were employed to determine the optimal threshold for predicting the efficacy of inosine in subgroup exploratory analysis. The hazard ratio (HR) and 95% confidence interval (95% CI) were estimated using a Cox proportional hazards model. A two‐sided *p*‐value <.05 indicated statistical significance for the observed differences. Statistical analyses were performed using SPSS version 26.0.0 and R version 4.0.2.

## 3 RESULTS

### 3.1 Participant characteristics

A total of 172 patients were enrolled from January 2021 to December 2022, with 86 patients each in the inosine and non‐inosine groups. The cutoff date for data statistics was September 30, 2023. There was no significant intergroup difference in the baseline information of the inosine and non‐inosine groups (Table 1).

**TABLE 1:** Intergroup comparison of the baseline information of the participants.

	Inosine group *N* (%) (*N* = 86)	Non‐inosine group *N* (%) (*N* = 86)	*χ* ^2^	*p*
Age, years				
<65	51 (59.3%)	43 (50.0%)	1.501	0.220
≥65	35 (40.7%)	43 (50.0%)		
ECOG score				
0	28 (32.6%)	31 (36.0%)	0.232	0.630
≥1	58 (67.4%)	55 (64.0)		
Sex				
Male	59 (68.6%)	49 (57.0%)	2.488	0.115
Female	27 (31.4%)	37 (43.0%)		
Smoking status				
Smoking	37 (43.0%)	44 (51.1%)	1.143	0.285
Non‐smoking	49 (57.0%)	42 (48.8%)		
Drinking status				
Drinking	20 (23.3%)	29 (33.7%)	2.312	0.128
Teetotaler	66 (76.7%)	57 (66.3%)		
Regimens			7.874	0.251
PD‐1/PD‐L1 inhibitor + paclitaxel for injection (albumin‐bound) + platinum drugs	26 (30.2%)	18 (21.0%)	1.955	0.162
PD‐1/PD‐L1 inhibitor + oxaliplatin injection + capecitabine tablets/tegafur–gimeracil–oteracil potassium capsule	9 (10.5%)	13 (15.1%)	0.834	0.361
PD‐1/PD‐L1 inhibitor + anlotinib hydrochloride capsules/lenvatinib mesylate capsules	10 (11.6%)	8 (9.3%)	0.248	0.618
PD‐1/PD‐L1 inhibitor + CE/EP	9 (10.5%)	3 (3.5%)	3.225	0.073
PD‐1/PD‐L1 inhibitor	5 (5.8%)	10 (11.6%)	1.826	0.177
PD‐1/PD‐L1 inhibitor + gemcitabine hydrochloride for injection + platinum drugs	4 (4.7%)	5 (5.8%)	0.117	0.732
Others	23 (26.7%)	29 (33.7%)	0.992	0.319
Chemotherapy lines			3.487	0.322
First	56 (65.1%)	45 (52.3%)	2.902	0.088
Second	19 (22.1%)	25 (29.1%)	1.099	0.294
Three	6 (7.0%)	11 (12.8%)	1.632	0.201
Fourth and higher	5 (5.8%)	5 (5.8%)	0.000	1.000
Tumor type			8.290	0.406
Non‐small cell lung cancer	16 (18.6%)	18 (20.9%)	0.147	0.702
Small cell carcinoma of the lung	15 (17.4%)	8 (9.3%)	2.459	0.117
Stomach cancer	11 (12.8%)	15 (17.4%)	0.725	0.395
Head and neck cancer	9 (10.5%)	6 (7.0%)	0.657	0.418
Pancreaticobiliary duct cancer	7 (8.1%)	5 (5.8%)	0.358	0.549
Colon cancer	4 (4.7%)	6 (7.0%)	0.425	0.515
Esophageal cancer	12 (14.0%)	7 (8.1%)	1.479	0.224
Ovarian cancer + cervical cancer + endometrial cancer	4 (4.7%)	5 (5.8%)		1.000
Others	8 (9.3%)	16 (18.6%)	3.099	0.122
Transfer organs			4.117	0.533
Brain	5 (5.8%)	13 (15.1%)	3.971	0.079
Liver	21 (24.4%)	22 (25.6%)	0.031	0.861
Lymph nodes	69 (80.2%)	73 (84.9%)	0.646	0.422
Lung	16 (18.6%)	20 (23.3%)	0.562	0.453
Bone	10 (11.6%)	18 (20.9%)	2.730	0.098
Adrenal	5 (5.8%)	6 (7.0%)	0.097	0.755
Number of distant metastasis to organs			3.400	0.183
0	27 (31.4%)	17 (19.8%)	3.054	0.081
1	27 (31.4%)	28 (32.6%)	0.027	0.870
≥2	32 (37.2%)	41 (47.7%)	1.928	0.165
TNM			3.745	0.154
III	14 (16.3%)	9 (10.5%)	1.255	0.263
IV	63 (73.3%)	73 (84.9%)	3.513	0.061
Others	9 (10.5%)	4 (4.7%)	2.080	0.149

Abbreviations: CE, etoposide injection + carboplatin; ECOG, Eastern Cooperative Oncology Group; EP, etoposide injection + cisplatin.

### 3.2 Outcomes

**FIGURE 1:**
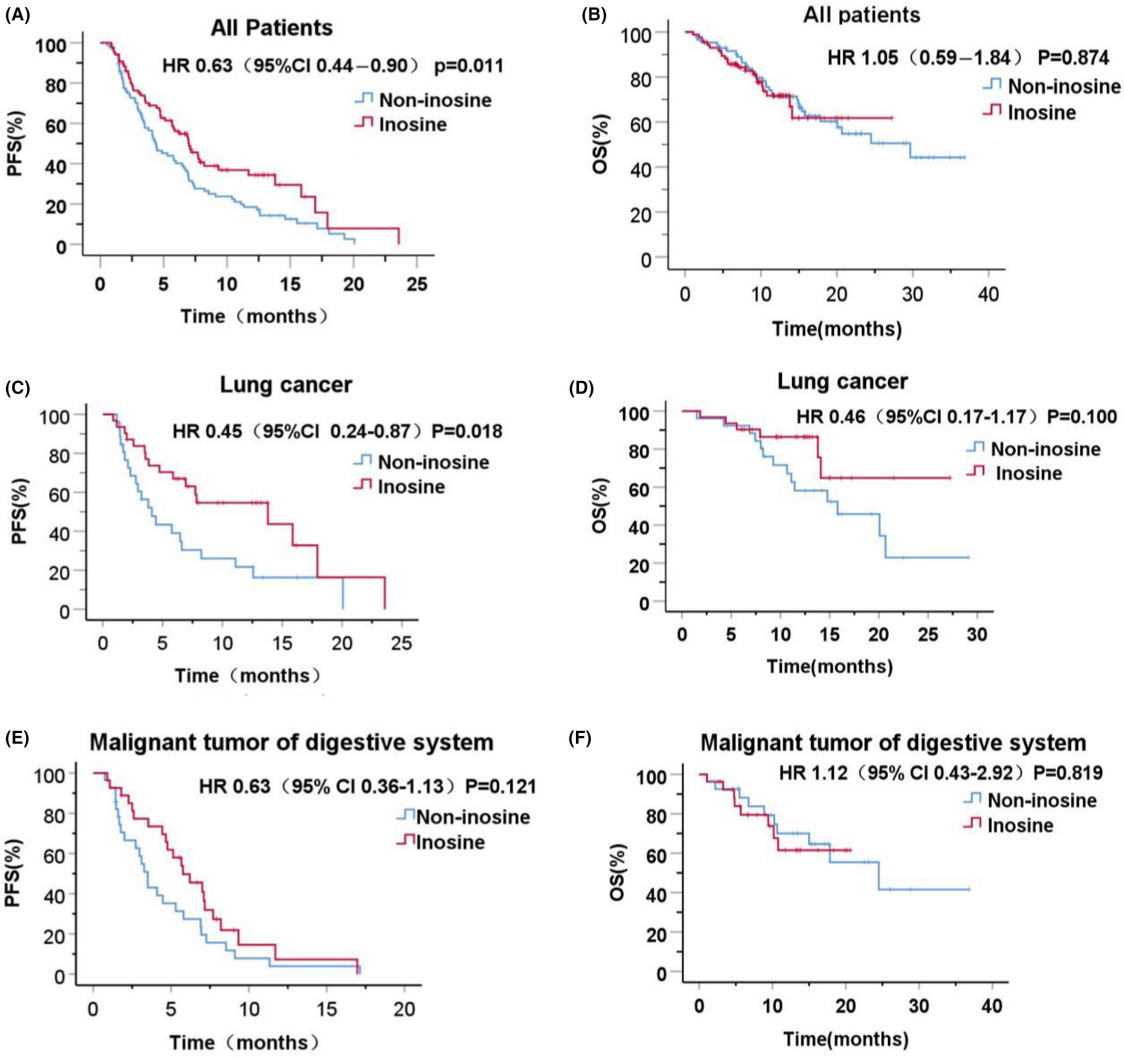
Kaplan–Meier method for estimating progression‐free survival (PFS) and overall survival (OS) for the inosine and non‐inosine groups. (A) PFS in the total study population. (B) OS in the total study population. (C) PFS in the lung cancer group. (D) OS in the lung cancer group. (E) PFS results in gastrointestinal malignancies. (F) OS results in gastrointestinal malignancies. HR, hazard ratio, 95% CI, 95% confidence interval.

As of September 30, 2023, all participants had completed at least one efficacy evaluation. In the inosine and non‐inosine groups (Table 2), the ORR was 26.74% and 15.12% (*p* = 0.061), whereas the DCR was 88.37% and 81.39% (*p* = 0.202). The median PFS (mPFS) was 7.00 (95% CI 5.31–8.69) and 4.40 (95% CI 3.10–5.70) months (HR 0.63; 95% CI 0.44–0.90, *p* = 0.011), respectively. Compared with the control group, the 6‐ and 12‐month PFS rates were significantly higher in the inosine group (Figure 1A; Table 2). The median OS (mOS) was not reached in the inosine group and was 29.67 months (95% CI 17.40–41.94) in the non‐inosine group (HR 1.05, 95% CI 0.59–1.84, *p* = 0.874). The 6‐ and 12‐month OS rates were 85.0% and 71.7% in the inosine group, as compared to 91.6% and 71.3% in the non‐inosine group (*p* = 0.205 and *p* = 0.723, respectively; Figure 1B; Table 2).

**TABLE 2:** Intergroup comparison of efficacy indicators.

	Inosine (*N* = 86)	Non‐inosine (*N* = 86)	*p*
CR *n* (%)	0 (0%)	0 (0%)	
PR *n* (%)	23 (26.74%)	13 (15.12%)	
SD *n* (%)	53 (61.62%)	57 (66.27%)	
PD *n* (%)	10 (11.62%)	16 (18.60%)	
ORR *n* (%)	23 (26.74%)	13 (15.12%)	0.061
DCR *n* (%)	76 (88.37%)	70 (81.39%)	0.202
PFS			
Events *n* (%)	54 (66.7%)	75 (87.0%)	
mPFS (95%CI)	7.00 m (5.31–8.69)	4.40 m (3.10–5.70)	0.011
6 mPFS (%)	56.1%	40.2%	0.047
12 mPFS (%)	34.4%	17.1%	0.006
OS			
Events *n* (%)	22 (25.6%)	32 (37.2%)	
mOS (95%CI)	NR	29.67 m (17.40–41.94)	0.874
6 mOS (%)	85.7%	91.6%	0.205
12 mOS (%)	71.7%	71.3%	0.723

Abbreviations: 12 mOS rate, 12‐month overall survival; 12 mPFS rate, 12‐month progression‐free survival; 6 mOS rate, 6‐month overall survival; 6 mPFS rate, 6‐month progression‐free survival; 95% CI, 95% confidence interval; CR, complete response; DCR, disease control rate; m, months; mOS, median overall survival; mPFS, median progression‐free survival; NR, not reached; ORR, objective response rate; OS, overall survival; PD, progression disease; PFS, progression‐free survival; PR, partial response; SD, stable disease.

In subgroup exploratory analysis among patients with lung cancer, 31 patients were included in the inosine group, of whom 17 (54.8%) experienced disease progression and 6 (19.4%) patients died. Similarly, a total of 26 patients were included in the non‐inosine group, of whom 21 (80.8%) experienced disease progression and 14 (53.8%) died. In the inosine and non‐inosine groups, the mPFS was 13.80 (95% CI 4.07–23.53) and 4.10 (95% CI 2.28–5.93) months, respectively (HR 0.45, 95% CI 0.24–0.87, *p* = 0.018; Figure 1C; Appendix S2: Table S2). Although mOS was not achieved in the inosine group, the mOS was 15.80 (95% CI 7.85–23.76) months in the non‐inosine group (HR 0.46, 95% CI 0.17–1.17, *p* = 0.100; Figure 1D; Appendix S2: Table S2).

In a subgroup exploratory analysis among participants with malignancies of the digestive system, of the 27 participants included in the inosine group, 22 (81.5%) experienced disease progression and 8 (29.6%) died. Similarly, of the 28 participants included in the non‐inosine group, 26 (92.9%) experienced disease progression and 10 (35.7%) died. The mPFS was 5.77 (95% CI 3.63–7.90) and 3.50 (95% CI 2.85–4.15) months in the inosine and non‐inosine groups, respectively (HR 0.63, 95% CI 0.36–1.13, *p* = 0.121; Figure 1E; Appendix S2: Table S3). Participants in the inosine group did not attain the mOS, whereas it was 24.50 (95% CI 11.63–37.37) months in the non‐inosine group (HR 1.12, 95% CI 0.43–2.92, *p* = 0.819; Figure 1F; Appendix S2: Table S3).

### 3.3 Survival and effective analysis in exploratory subgroups

Compared with the non‐inosine group, the inosine group showed superior mPFS in subgroups with platelet–lymphocyte ratio (PLR) <229, neutrophil–lymphocyte ratio (NLR) <4, lactate dehydrogenase (LDH) ≥216, eosinophil–neutrophil ratio (ENR) ≤0.012, non‐drinking, smokers, ECOG ≥1, metastasis, no adrenal metastasis, no bone metastasis, no lung metastasis, lymphatic metastasis, liver metastasis, no brain metastasis, brain metastasis, and Stage IV, with significant differences. The mOS did not differ significantly between the subgroups (Figure 2; Appendix S1: Figures S1–S19; Appendix S2: Tables S4–S22).

**FIGURE 2:**
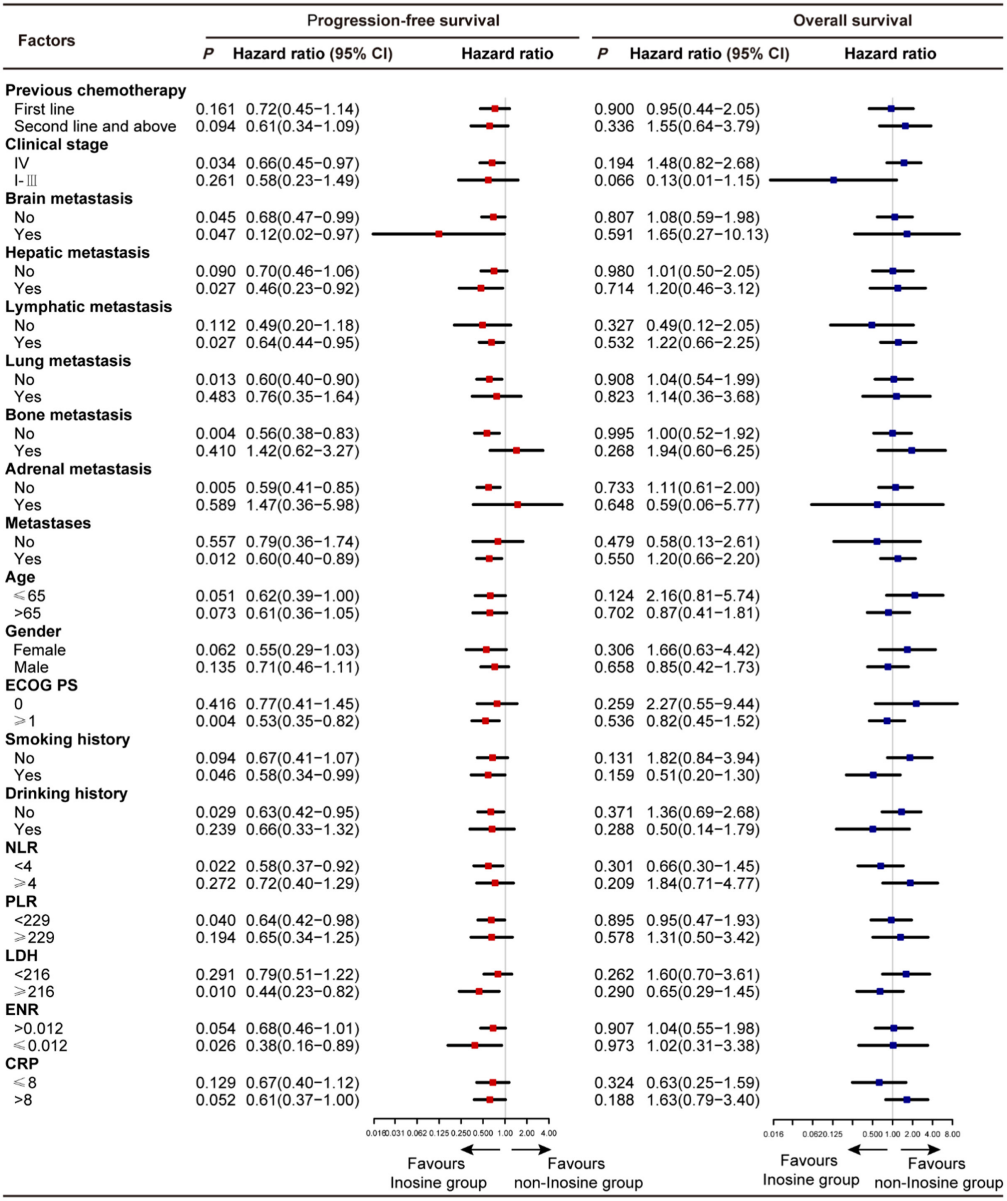
Forest plots of overall and progression‐free survival in patient subgroups. CRP, C‐reactive protein; ECOG, Eastern Cooperative Oncology Group; ENR, eosinophil–neutrophil ratio; LDH, lactate dehydrogenase; NLR, neutrophil–lymphocyte ratio; PLR, platelet–lymphocyte ratio.

Among the subgroups stratified by laboratory results, in the inosine and non‐inosine groups, respectively, the mPFS was 7.10 (95% CI 5.89–8.31) and 4.47 (95% CI 2.76–6.18) months for the PLR < 229 group (HR 0.64, 95% CI 0.42–0.98, *p* = 0.040); 7.10 (95% CI 6.11–8.09) and 4.33 (95% CI 2.89–5.77) months for the NLR <4 group (HR 0.58, 95% CI, 0.37–0.92, *p* = 0.022); 7.10 (95% CI 3.78–10.42) and 2.93 (95% CI 1.23–4.63) months for the LDH≥216 group (HR 0.44, 95% CI 0.23–0.82, *p* = 0.010); and 5.88 (95% CI 5.55–6.19) and 3.33 (95% CI 2.86–3.81) months for the ENR≤0.012 group (HR 0.38, 95% CI 0.16–0.89, *p* = 0.026).

Among the subgroups stratified by baseline characteristics, in the inosine and non‐inosine groups, respectively, the mPFS was 7.00 (95% CI 5.93–8.05) and 4.43 (95% CI 2.72–6.15) months for the non‐drinking group (HR 0.63, 95% CI 0.42–0.95, *p* = 0.029); 7.10 (95% CI 3.41–10.79) and 4.23 (95% CI 1.43–7.04) for the smoking group (HR 0.58, 95% CI 0.34–0.99, *p* = 0.046); and 6.87 (95% CI 5.52–8.21) and 4.10 (95% CI 2.78–5.42) months for the ECOG≥1 group (HR 0.53, 95% CI 0.35–0.82, *p* = 0.004).

Among the subgroups stratified by the site of metastasis, in the inosine and non‐inosine groups, respectively, the mPFS was 6.97 (95% CI, 5.14–8.80) and 4.23 (95% CI, 3.31–5.16) months for the no adrenal metastasis group (HR 0.59, 95% CI 0.41–0.85, *p* = 0.005); 7.70 (95% CI 6.56–8.84) and 4.13 (95% CI 3.11–5.16) for the no bone metastasis group (HR 0.56, 95% CI 0.38–0.83, *p* = 0.004); 7.00 (95% CI 5.59–8.41) and 4.23 (95% CI 3.31–5.16) months for the no lung metastasis group (HR 0.60, 95% CI 0.40–0.90, *p* = 0.013); 6.97 (95% CI 3.67–10.27) and 2.77 (95% CI 0.20–5.33) months for the hepatic metastasis group (HR 0.46, 95% CI 0.23–0.92, *p* = 0.027); 6.93 (95% CI 5.66–8.21) and 4.33 (95% CI 2.95–5.73) months for the no brain metastasis group (HR 0.68, 95% CI 0.47–0.99, *p* = 0.045); 13.80 (NR) and 4.43 (95% CI 0.38–8.49) months for the brain metastasis group (HR 0.12, 95% CI 0.02–0.97, *p* = 0.047); 6.93 (95% CI 5.46–8.40) and 4.43 (95% CI 3.22–5.65) months for the Stage IV group (HR 0.66, 95% CI 0.45–0.97, *p* = 0.034); 6.93 (95% CI 5.03–8.84) and 4.43 (95% CI 2.72–6.15) months for the lymphatic metastasis group (HR 0.64, 95% CI 0.44–0.95, *p* = 0.027); and 6.97 (95% CI 5.60–8.33) and 4.40 (95% CI 2.95–5.85) months for the metastasis group (HR 0.61, 95% CI 0.40–0.89, *p* = 0.012).

Further exploratory subgroup analyses showed that the ORR significantly improved in the inosine group compared with that of the non‐inosine group in the non‐drinking, non‐smoking, age >65 years, no adrenal metastasis, no bone metastasis, lung metastasis, or brain metastasis groups (Appendix S2: Table S1).

### 3.4 Univariate and multivariate Cox regression analysis based on PFS and OS

Univariate Cox regression analysis was performed for PFS and OS in all patients and the inosine groups; subgroups with univariate Cox results of *p* < 0.100 were selected for multifactorial Cox regression analysis.

#### 3.4.1 Whole cohort (Inosine+Non‐inosine)

The results of the univariate analysis showed that the number of lines, ENR, and inosine treatment were associated with PFS in all patients, whereas aging, brain metastasis, liver metastasis, bone metastasis, metastasis, age, ECOG, LDH, and NLR were associated with OS in all patients.

**FIGURE 3:**
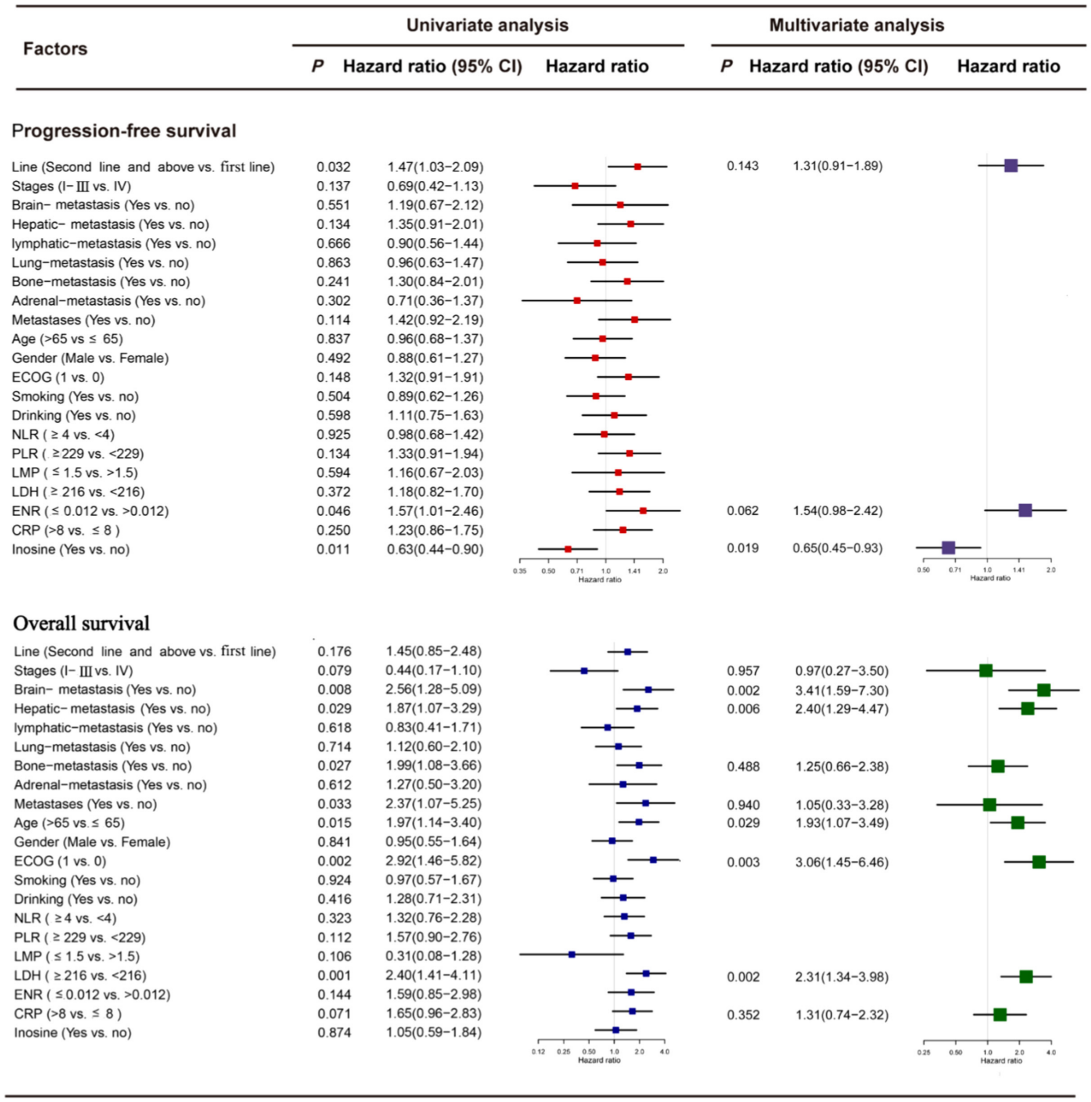
Univariate and multivariate Cox regression analysis of the all patients, with regard to the PFS and OS. OS, overall survival; PFS, progression‐free survival.

The multifactorial analysis revealed that the inosine treatment group exhibited reduced risk of disease progression compared to the non‐inosine treatment group (HR 0.65, 95% CI 0.45–0.93, *p* = 0.019). Regarding OS, the mortality risk increased in the brain metastasis group compared with the no brain metastasis (HR 3.41, 95% CI, 1.59–7.30, *p* = 0.002), in the hepatic metastasis group compared with the no hepatic metastasis group (HR 2.40, 95% CI 1.29–4.47, *p* = 0.006), in the age >65 years group compared with the age ≤65 years group (HR 1.93, 95% CI 1.07–3.49, *p* = 0.029), in the ECOG≥1 group compared with the ECOG = 0 group (HR 3.06, 95% CI 1.45–6.46, *p* = 0.003); and in the LDH≥216 group compared with the LDH < 216 group (HR 2.31, 95% CI 1.34–3.98, *p* = 0.002) (Figure 3).

#### 3.4.2 Inosine group

**FIGURE 4:**
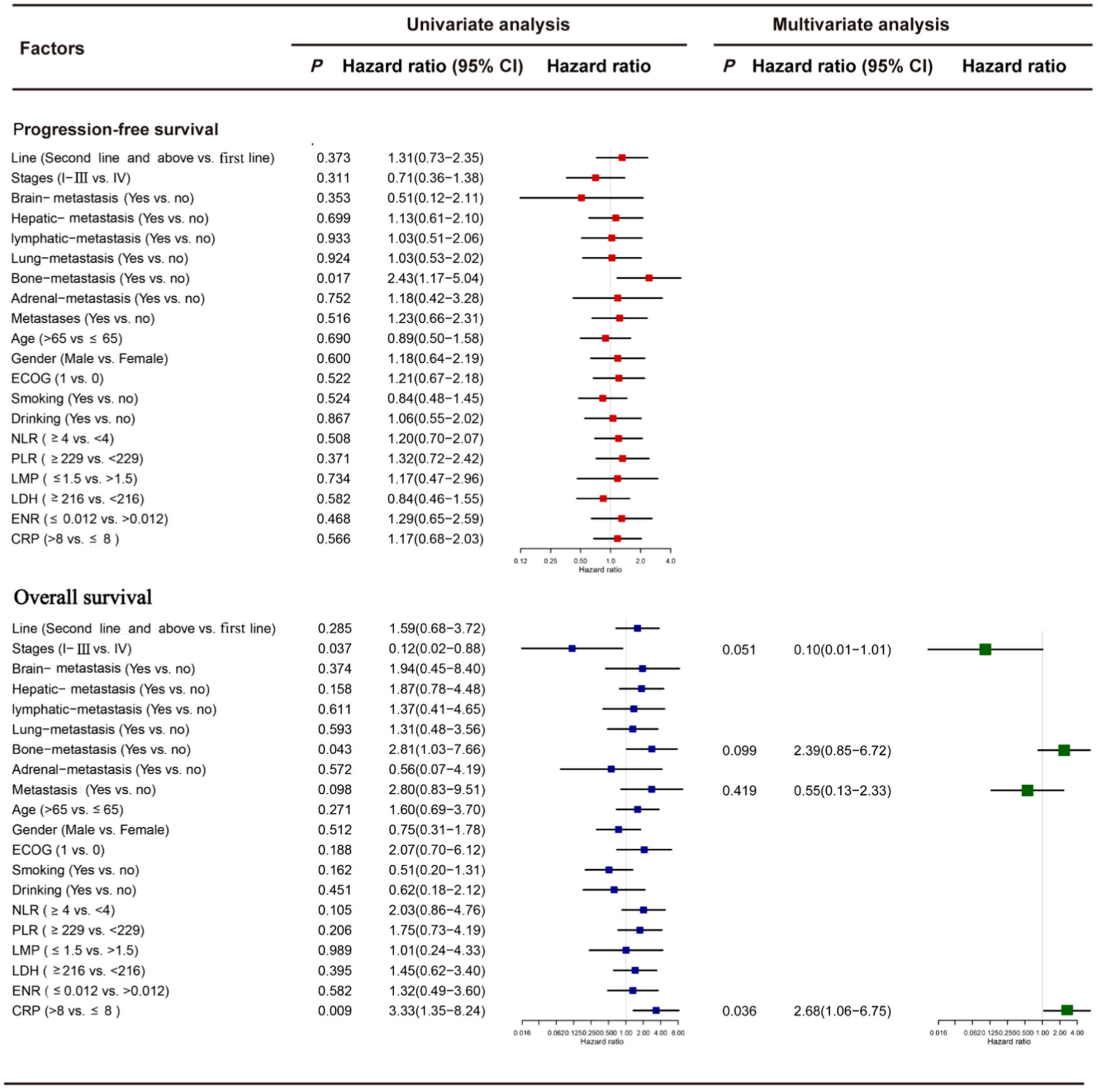
Univariate and multivariate Cox regression analysis of the inosine group, with regard to the PFS and OS. OS, overall survival; PFS, progression‐free survival.

Univariate analysis showed that bone metastasis was associated with PFS and that staging, bone metastasis, and CRP were associated with OS, in the inosine group. Multifactorial analysis showed that CRP >8 increased the mortality risk compared with CRP≤8 (HR 2.68, 95% CI 1.06–6.75, *p* = 0.036; Figure 4).

### 3.5 Safety analysis

Adverse events were graded according to CTCAE version 5.0. The association of adverse events with the drug according to the five‐point method in this trial, they were considered related to the study drug with relevance, very likely relevance, and possible relevance and unrelated to the study drug with possibly unrelated and unrelated. The Grades 3–4 adverse reactions in the inosine group included neutropenia in 8 (9%) cases, platelet decrease in 10 (12%) cases, alanine aminotransferase increased in 3 (3%) cases, aspartate aminotransferase increased in 4 (5%) cases. The Grades 3–4 adverse reactions in the non‐inosine group included neutropenia in 11 (13%) patients, platelet decrease in 11 (13%) patients, alanine aminotransferase increased in 4 (5%) patients, and aspartate aminotransferase increase in five (6%) patients. No Grade 5 adverse reactions were observed in either group. All Grades 3 and 4 adverse reactions observed were stratified as follows: 25 (29%) and 31 (36%) in the inosine and non‐inosine groups, respectively (*p* = 0.329; Table 3).

**TABLE 3:** All adverse reactions in both the study groups.

Adverse reactions	Inosine (*N* = 86)		Non‐inosine (*N* = 86)	
	All Grades	Grades 3–4	All Grades	Grades 3–4
Neutropenia *n* (%)	49 (57%)	8 (9%)	60 (70%)	11 (13%)
Decreased platelet count, *n* (%)	29 (34%)	10 (12%)	34 (40%)	11 (13%)
Increased alanine aminotransferase, *n* (%)	18 (21%)	3 (3%)	16 (19%)	4 (5%)
Increased aspartate aminotransferase, *n* (%)	20 (23%)	4 (5%)	20 (23%)	5 (6%)
Increased bilirubin, *n* (%)	9 (10%)	0 (0%)	12 (14%)	0 (0%)
Myocardial injury, *n* (%)	2 (2%)	0 (0%)	2 (2%)	0 (0%)
Increased creatinine, *n* (%)	1 (1%)	0 (0%)	0 (0%)	0 (0%)
Rash, *n* (%)	1 (1%)	0 (0%)	1 (1%)	0 (0%)
Exacerbation of psoriasis, *n* (%)	1 (10%)	0 (0%)	0 (0%)	0 (0%)
Interstitial pneumonia, *n* (%)	0 (0%)	0 (0%)	2 (2%)	0 (0%)
Hypothyroidism, *n* (%)	1 (1%)	0 (0%)	2 (2%)	0 (0%)
Blurred vision, *n* (%)	0 (0%)	0 (0%)	1 (1%)	0 (0%)
Sore throat, *n* (%)	0 (0%)	0 (0%)	1 (1%)	0 (0%)

## 4 DISCUSSION

Several clinical trials have demonstrated the effectiveness of ICI at 10–30% in all cancer treatment (CheckMate 078,^37^ KEYNOTE‐189,^38^ KEYNOTE‐024,^17^, ^39^ KEYNOTE‐151,^40^ KEYNOTE‐181,^11^ and KEYNOTE‐177^41^). Inosine enhances the efficacy of ICI by modulating the intrinsic immune response, adaptive immune response, and tumor cell immunogenicity in the tumor immune microenvironment.^21^, ^32^, ^33^, ^34^, ^35^, ^36^, ^42^

Mager et al. administered a combination of inosine + CpG + anti‐CTLA‐4 in mouse models of colorectal, melanoma, and bladder cancer, all of which showed that inosine enhanced the antitumor immunity of anti‐CTLA‐4 in the presence of the co‐stimulatory factor CPG.^26^, ^43^ In mice with B16 melanoma, Wang et al. showed that compared to treatment with a single PD‐L1 inhibitor, an oral combination therapy regimen comprising inosine and PD‐L1 inhibitor delayed tumor growth and prolonged survival. This combination significantly increased the number of CD8^+^ tumor‐infiltrating T lymphocytes (TIL) expressing IFN‐γ and TNF‐α and resulted in a higher percentage of circulating CD8^+^ T cells expressing IFN‐γ and TNF‐α in the draining lymph nodes and the spleen.^36^ Inosine supplementation enhanced the efficacy and durability of T cell‐based cancer immunotherapy in a preclinical mouse model.^36^ Zhang et al. found that inosine enhanced the immunotherapeutic response, both in mice and humans, by acting on UBA6 of tumor cells, increasing the immunogenicity of tumor cells, and thus enhancing the killing effect of T cells.^35^

Nonetheless, only preclinical, but not clinical, reports have shown that the microbial metabolite inosine can enhance the therapeutic efficacy of ICI. In this study, the mPFS in the inosine group was prolonged by 2.60 months compared with the non‐inosine group, and inosine reduced the risk of disease progression by 37%, which was statistically significant. The mOS did not differ significantly; however, this was possibly related to several factors, including the subsequent treatment regimen and individual circumstances. The ORR in the inosine group was approximately 10% higher than in the non‐inosine group, and thus lacked statistical significance. Both mPFS and ORR were significantly prolonged and improved in multiple exploratory subgroup analyses. This clinical trial showed that inosine had a tendency to enhance the efficacy of ICIs and delay disease progression, and these findings are consistent with the results of previous animal and preclinical studies.

In terms of safety, Grades 3 and 4 adverse reactions were observed in 25 (29%) and 31 (36%) patients in the inosine and non‐inosine groups, respectively, and inosine likely reduced immunotherapy‐related adverse reactions. The results of this trial suggest that, compared with that in the non‐inosine group, the mPFS was significantly prolonged in the inosine group. Inosine combined with PD‐1/PD‐L1 inhibitors has a synergistic effect that will provide a new option for overcoming immunotherapy resistance. Inosine tablets have been used clinically as a long‐term adjuvant treatment for hepatitis, is safe and economical, and will not impose a huge economic burden on the patient. In the subgroup analysis, the median PFS for lung cancer and digestive system malignancies tended to increase in the inosine group. Future Phase III clinical trials should focus on the selection of a single tumor species of lung or gastrointestinal system cancer in an expanded sample size for the verification of these findings. OS is related to the patients' general status before enrollment, age, disease stage, number of previous treatment lines, follow‐up treatment opportunities, and the follow‐up treatment protocols after disease progression. Thus, future phase III clinical studies should balance these factors and provide a detailed assortment of these factors for subgroup analysis to more accurately determine the relationship between drugs and OS. Owing to the limited sample size of this trial, the single‐center design, enrolled multi‐kinds of cancers, and non‐pre‐designed subgroups. Therefore, the results of the trial have some limitations and require further validation in a multicenter clinical trial with a larger sample.

## 5 CONCLUSION

In this trial, the median PFS in the inosine group was 2.6 months longer than that in the non‐inosine group and the risk of disease progression was reduced by 37%. The median OS was not reached in the inosine group and was 29.67 months in the non‐inosine group. The ORR of the inosine group was higher than that of the non‐inosine group, although the difference was not statistically significant. Inosine had a tendency to enhance the efficacy of ICIs and reduced immunotherapy‐related adverse reactions in clinical applications.

## AUTHOR CONTRIBUTIONS

**Haiqing Zhao:** Data curation (lead); formal analysis (lead); methodology (lead); project administration (lead); writing – original draft (lead); writing – review and editing (lead). **Wei Zhang:** Data curation (equal); formal analysis (equal); writing – review and editing (equal). **Yuting Lu:** Software (supporting); writing – review and editing (supporting). **Yin Dong:** Data curation (supporting); project administration (supporting). **Zhihao He:** Conceptualization (supporting); data curation (supporting). **Hongchao Zhen:** Data curation (supporting); project administration (supporting). **Qin Li:** Conceptualization (supporting); data curation (supporting); formal analysis (supporting); funding acquisition (supporting); methodology (supporting); resources (supporting); supervision (supporting); writing – review and editing (supporting).

## FUNDING INFORMATION

This study was financially supported by the National Natural Science Foundation of China (Grant No. 82273282).

## CONFLICT OF INTEREST STATEMENT

The authors declare no competing interests.

## ETHICS STATEMENT

This trial was approved (ethical approval number: 2021‐P2‐152‐02) by the Clinical Investigation Ethics Committee, Beijing Friendship Hospital, Capital Medical University.

## INFORMED CONSENT

All patients provided written informed consent for data collection and study participation.

## CLINICAL TRIAL REGISTRATION

This trial is registered with ClinicalTrials.gov (NCT05809336).

## Supporting information


Appendix S1:

Appendix S2:

Appendix S3:

## Data Availability

The data supporting the findings of this study are available upon request from the corresponding author, Qin Li.
