# Supplementary material for: Inosine enhances the efficacy of immune‐checkpoint inhibitors in advanced solid tumors: A randomized, controlled, Phase 2 study
Source: Cancer Med. 2024 Sep 13;13(17):e70143. doi: 10.1002/cam4.70143 (PMC11393481; doi:10.1002/cam4.70143)
Supplement: Supplementary file 1 — Appendix S1: [file CAM4-13-e70143-s002.docx]

# Appendix 1- Figures

**Inosine Enhances the Efficacy of Immune-checkpoint Inhibitors in Advanced Solid Tumors: A Randomized, Controlled, Phase 2 Study**

Haiqing Zhao, Wei Zhang, Yuting Lu,Yin Dong, Zhihao He, Hongchao Zhen, Qin Li*

Inosine had a tendency to enhance the efficacy of ICIs and reduced immunotherapy-related adverse reactions in clinical applications.


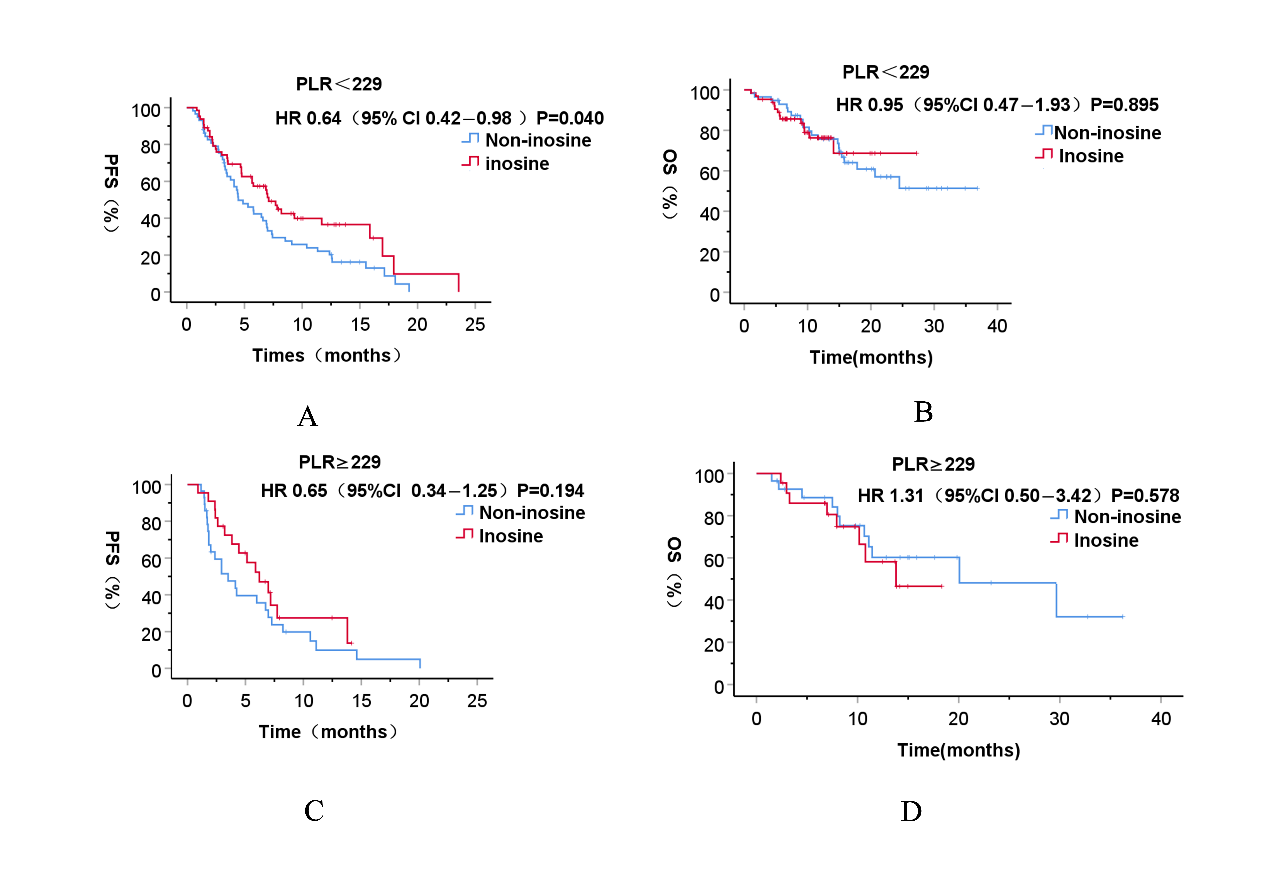


## Figure1.Progression-free survival (PFS) in patients of the PLR<229 (A) and patients of the PLR≥229 (C), overall survival (OS) in patients of the PLR<229 (B) and patients of the PLR≥229 (D). 95% CI=95% confidence interval, HR=hazard ratio, PLR=platelet to lymphocyte ratio


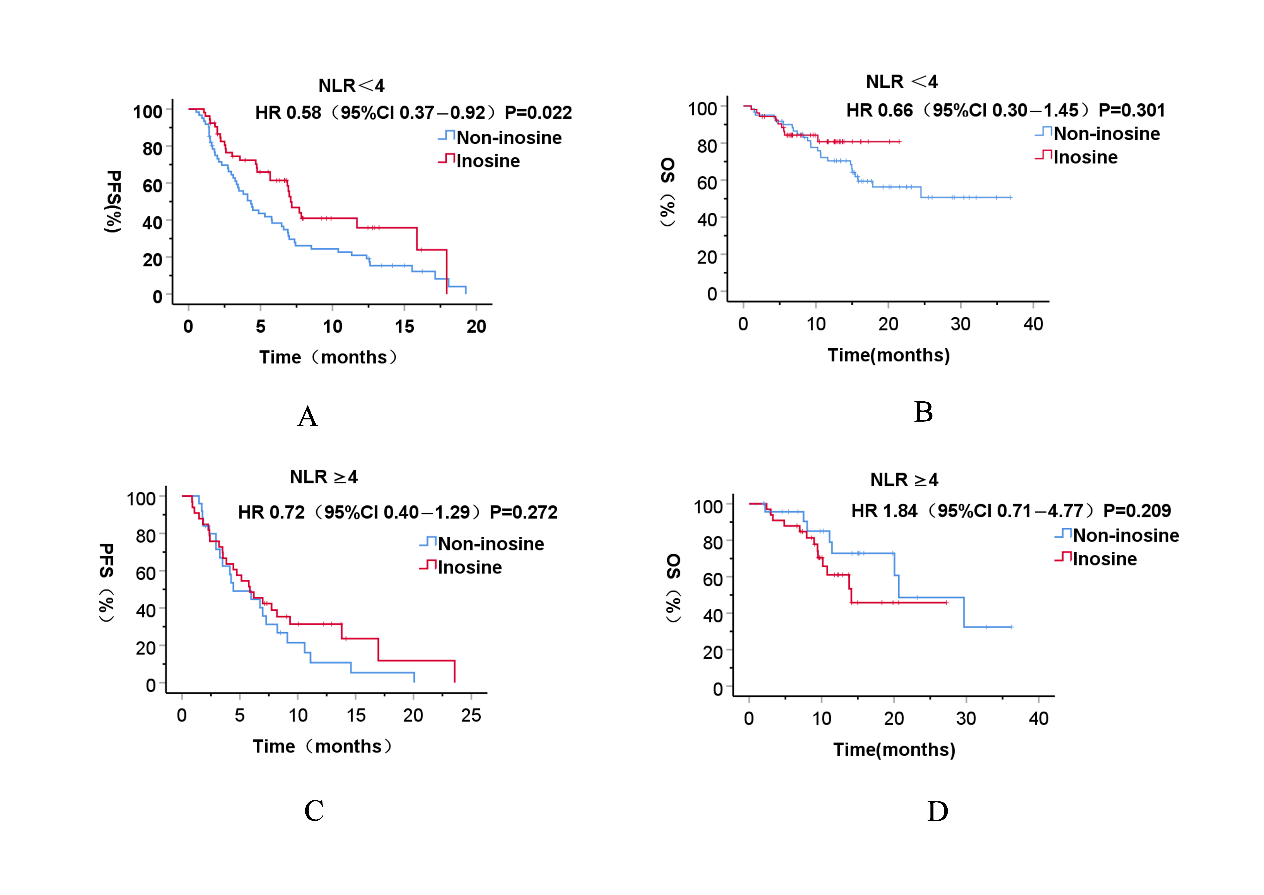


## Figure2. Progression-free survival (PFS) in patients of the NLR<4 (A) and patients of the NLR≥4(C), overall survival (OS) in patients of the NLR<4 (B) and patients of the NLR≥4(D). 95% CI=95% confidence interval, HR=hazard ratio, NLR=neutrophil to lymphocyte ratio.


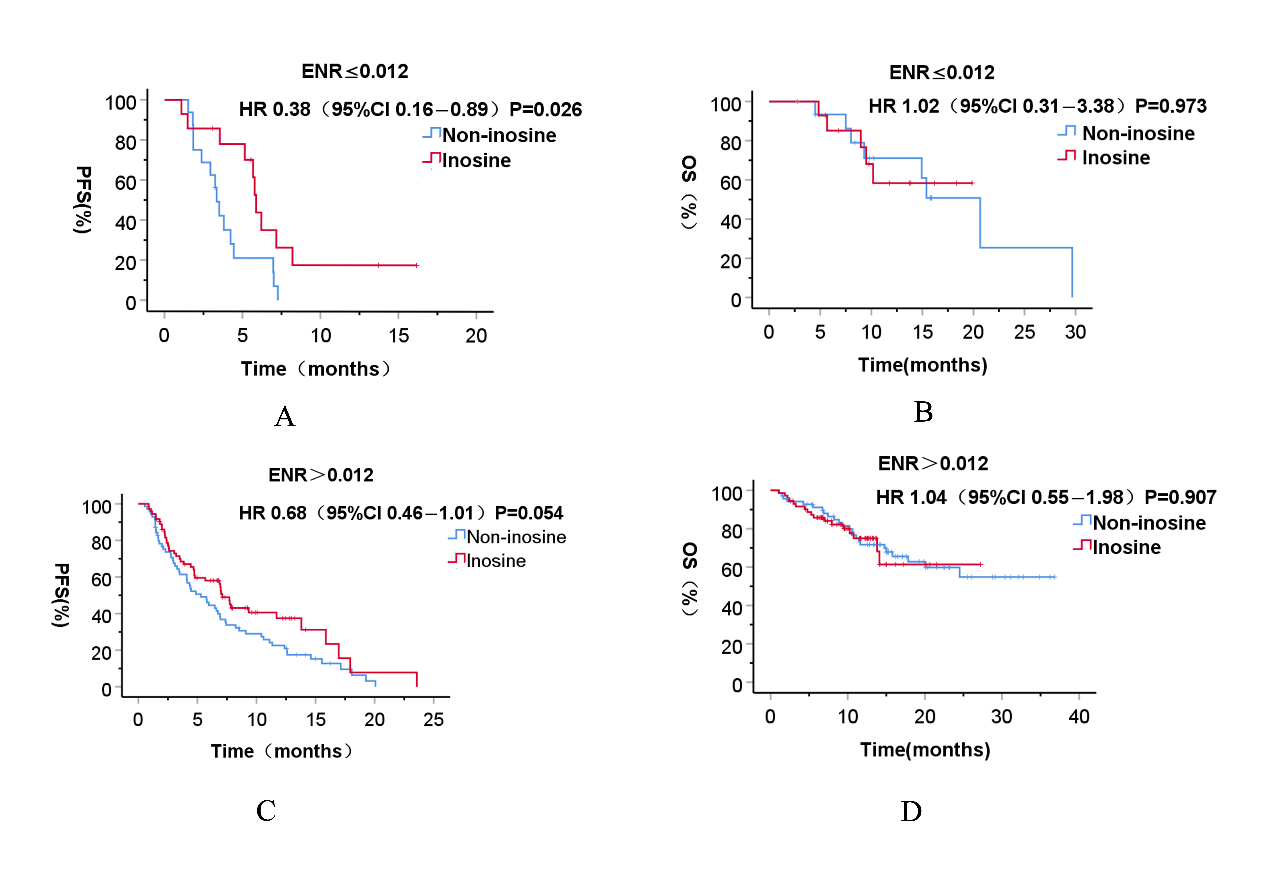


**Figure3.** Progression-free survival (PFS) in patients of the ENR≤0.012 (A) and patients of the ENR＞0.012 (C), overall survival (OS) in patients of the ENR≤0.012 (B) and patients of the ENR＞0.012(D). 95% CI=95% confidence interval, HR=hazard ratio, ENR=eosinophil-to-neutrophil ratio


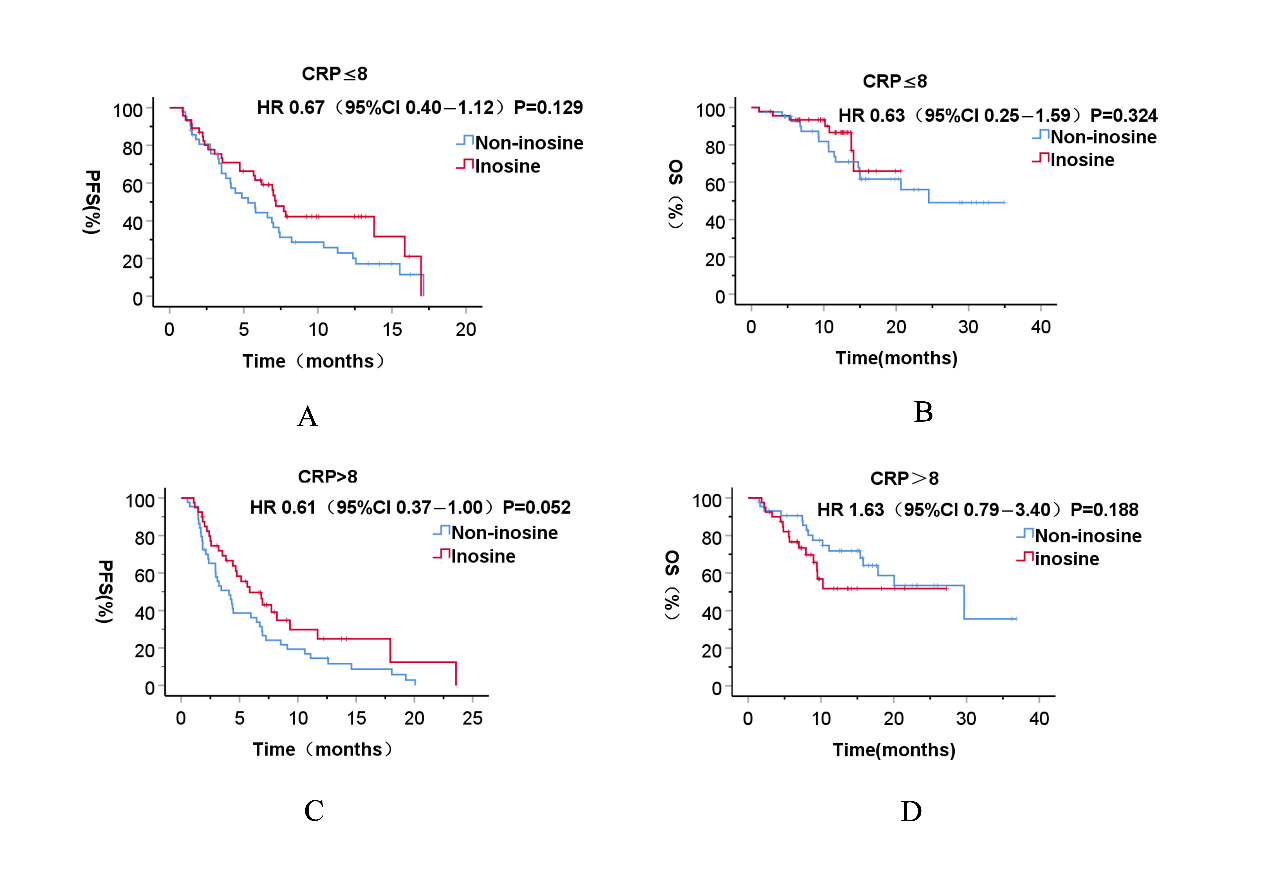


## Figure4. Progression-free survival (PFS) in patients of the CRP≤8 (A) and patients of the CRP＞8(C), overall survival (OS) in patients of the CRP≤8 (B) and patients of the CRP＞8 (D). 95%CI=95% confidence interval, HR=hazard ratio, CRP=C-Reactive protein.


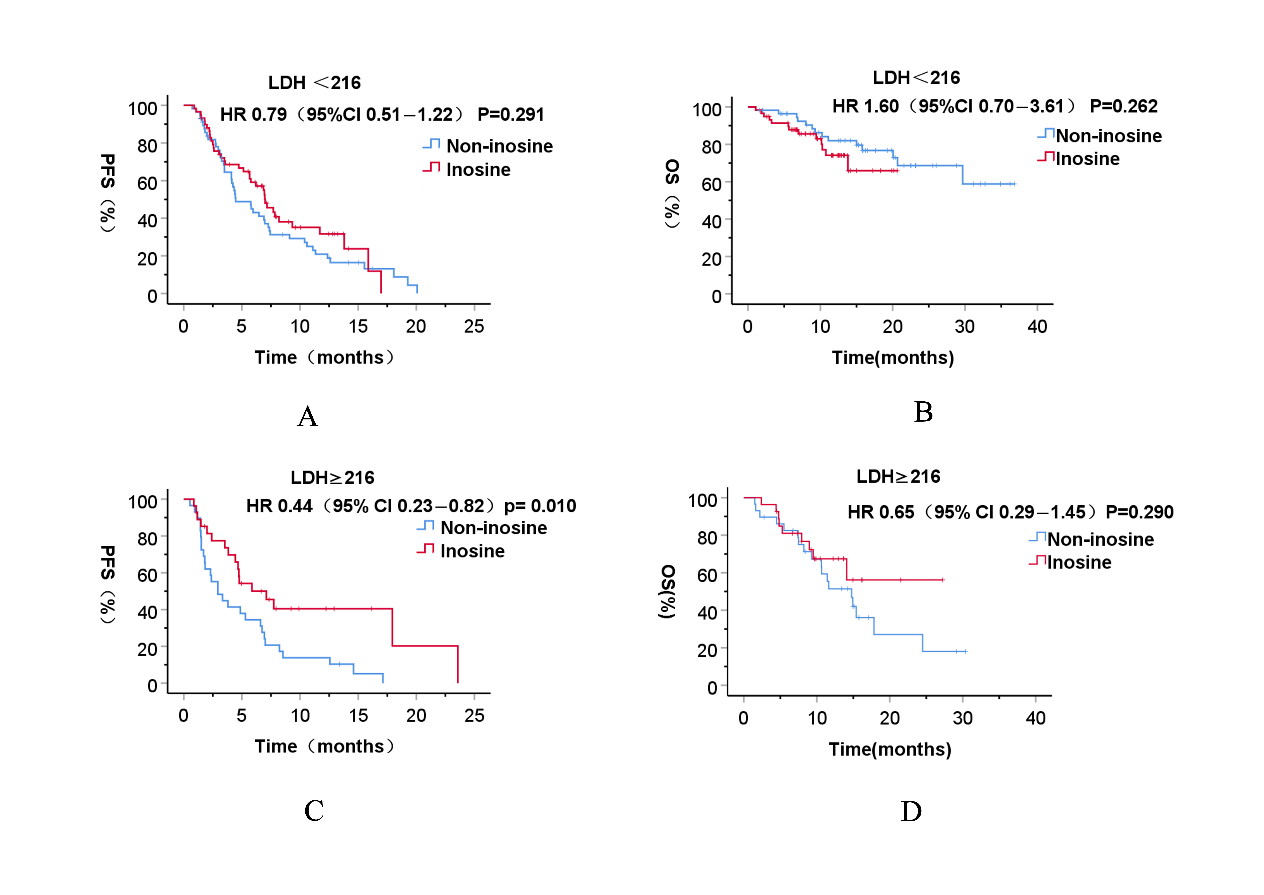


## Figure5. Progression-free survival (PFS) in patients of the LDH<216 (A) and patients of the LDH≥216 (C), overall survival (OS) in patients of the LDH<216 (B) and patients of the LDH≥216 (D), 95% CI=95% confidence interval, HR=hazard ratio, LDH=lactate dehydrogenase.


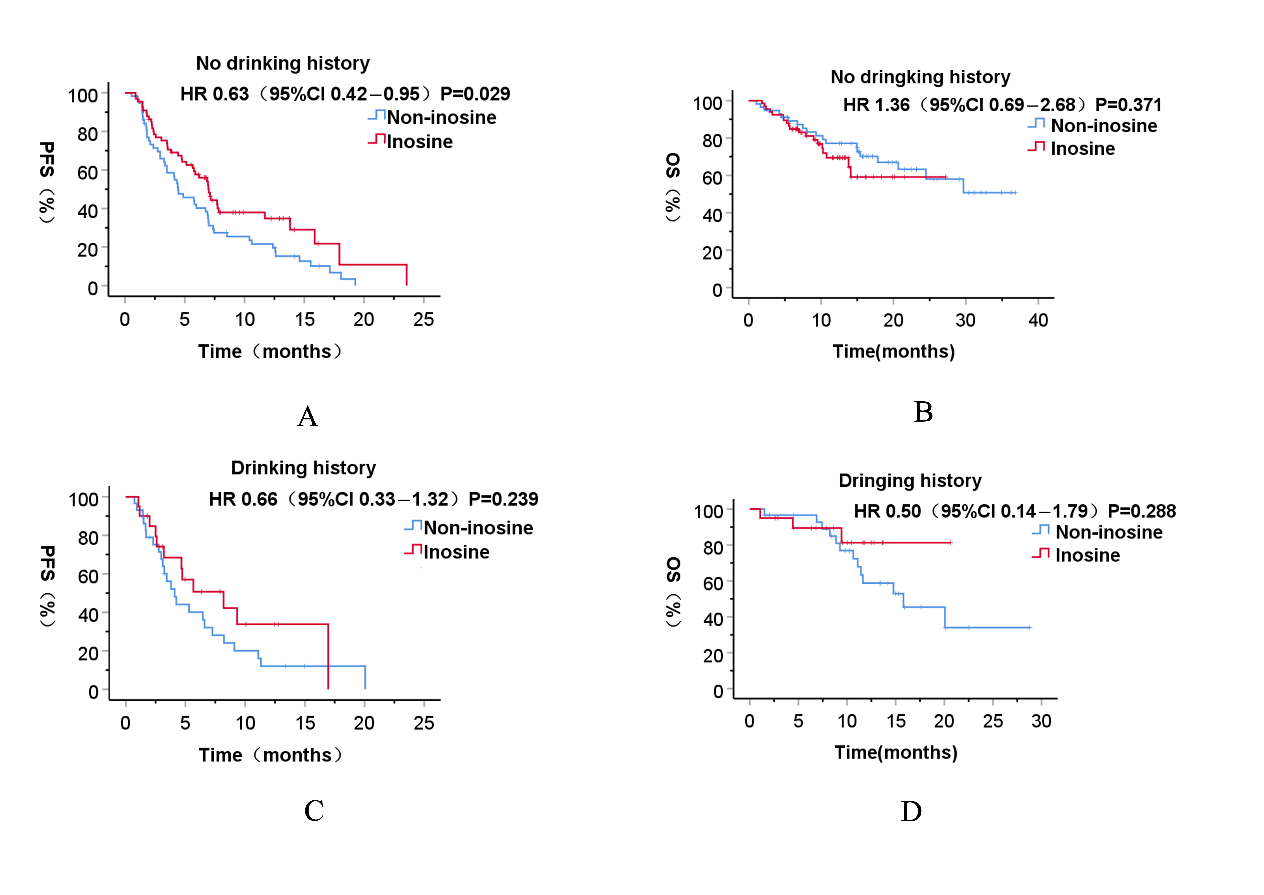


## Figure6. Progression-free survival (PFS) in patients of the non-drinking (A) and patients of the drinking (C), overall survival (OS) in patients of the no drinking (B) and patients of the drinking (D). 95% CI=95% confidence interval, HR=hazard ratio.


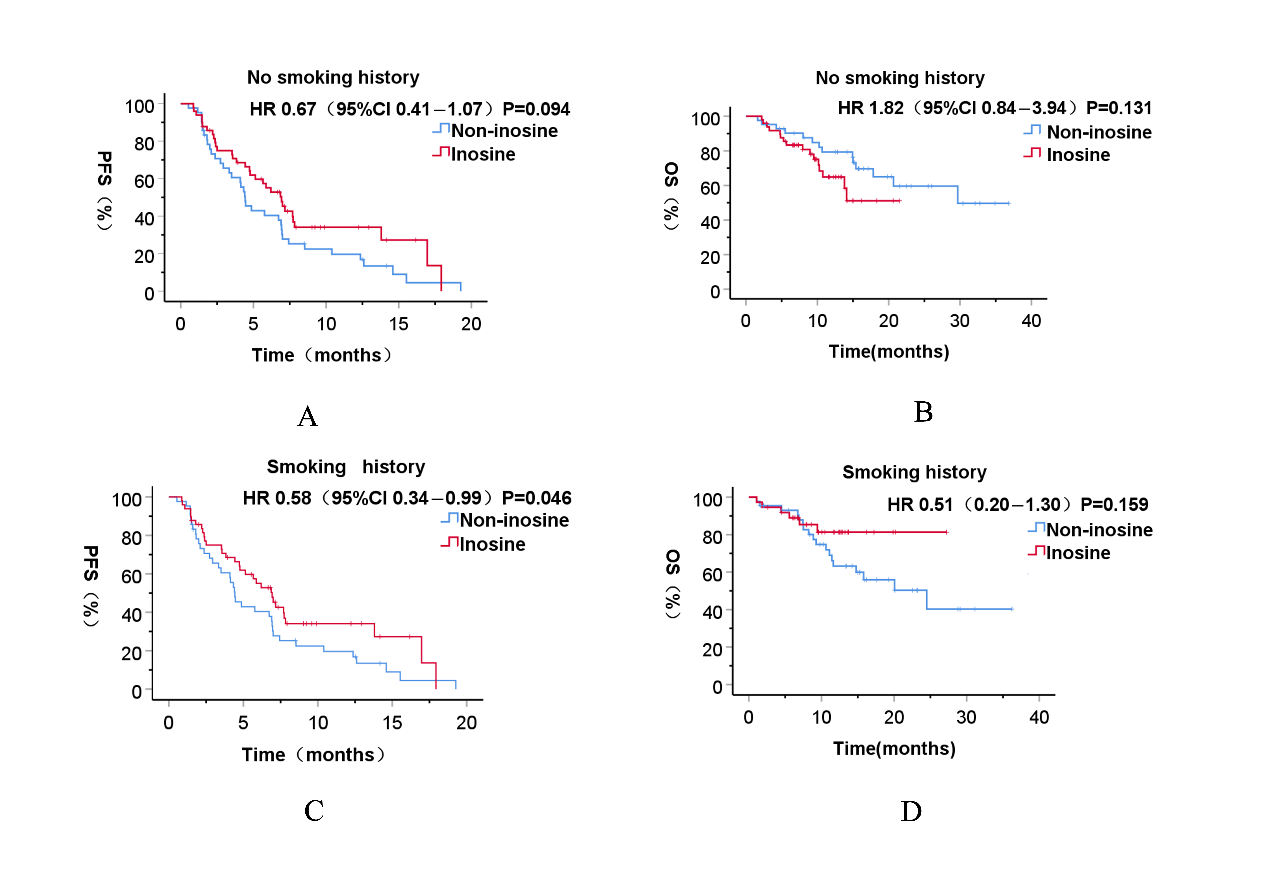


## Figure7. Progression-free survival (PFS) in patients of the non-smoking (A) and patients of the smoking (C), overall survival (OS) in patients of the no smoking (B) and patients of the smoking (D), 95% CI=95% confidence interval, HR=hazard ratio.


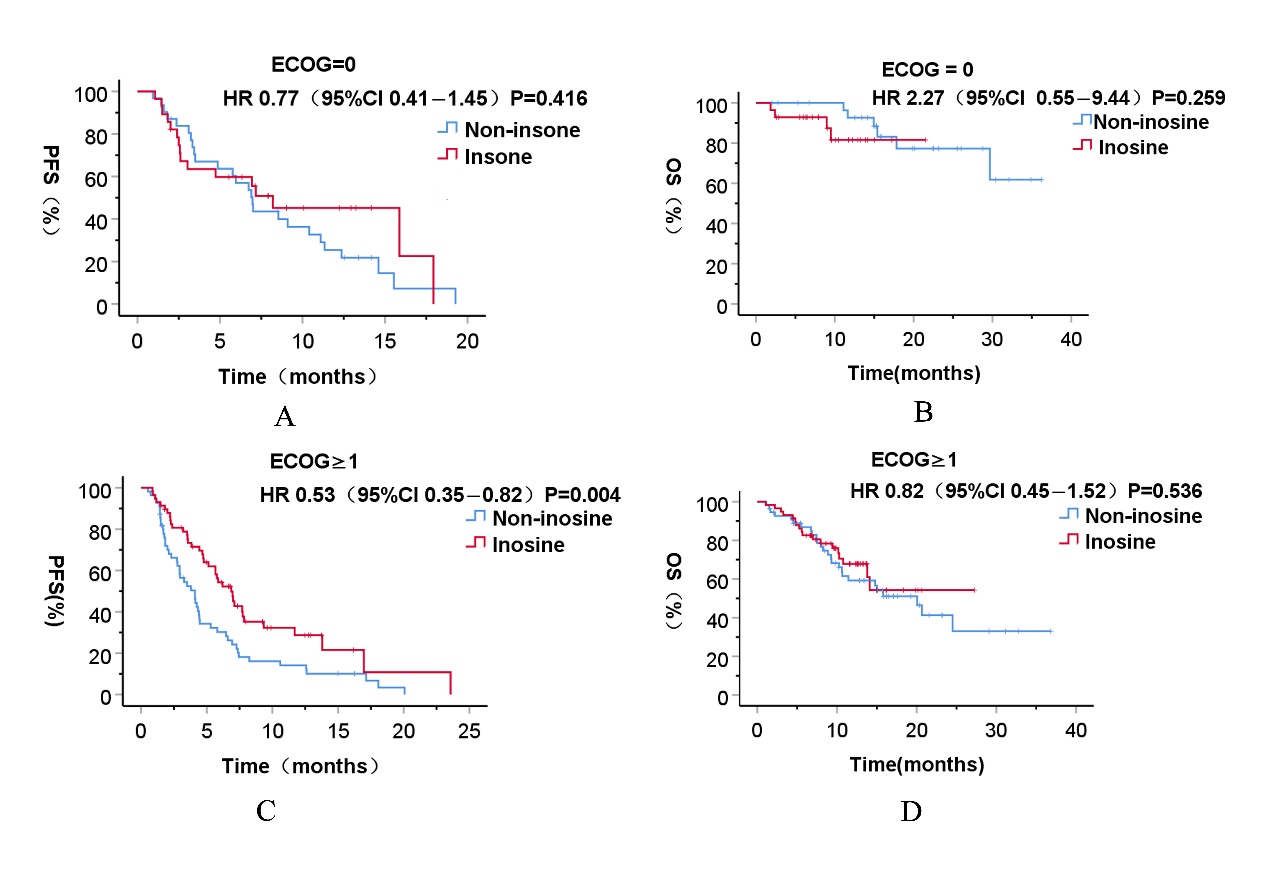


## Figure8.Progression-free survival (PFS) in patients of the ECOG=0 (A) and patients of the ECOG≥1(C), overall survival (OS) in patients of the ECOG=0 (B) and patients of the ECOG≥1 (D). 95% CI=95% confidence interval, HR=hazard ratio, ECOG=Eastern Cooperative Oncology Group.


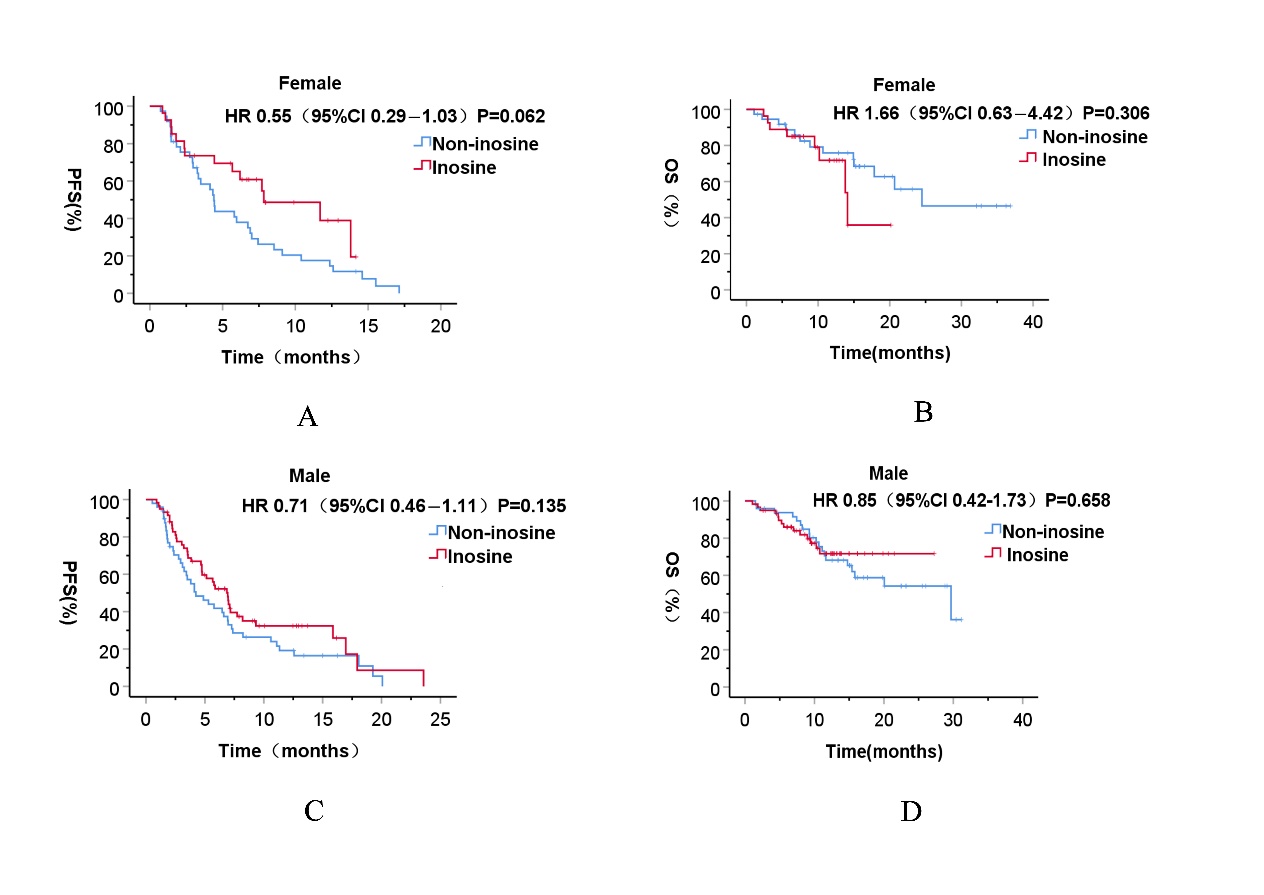


## Figure9.Progression-free survival (PFS) in patients of the female (A) and patients of the male (C), overall survival (OS) in patients of the female (B) and patients of the male (D). 95% CI=95% confidence interval, HR=hazard ratio.


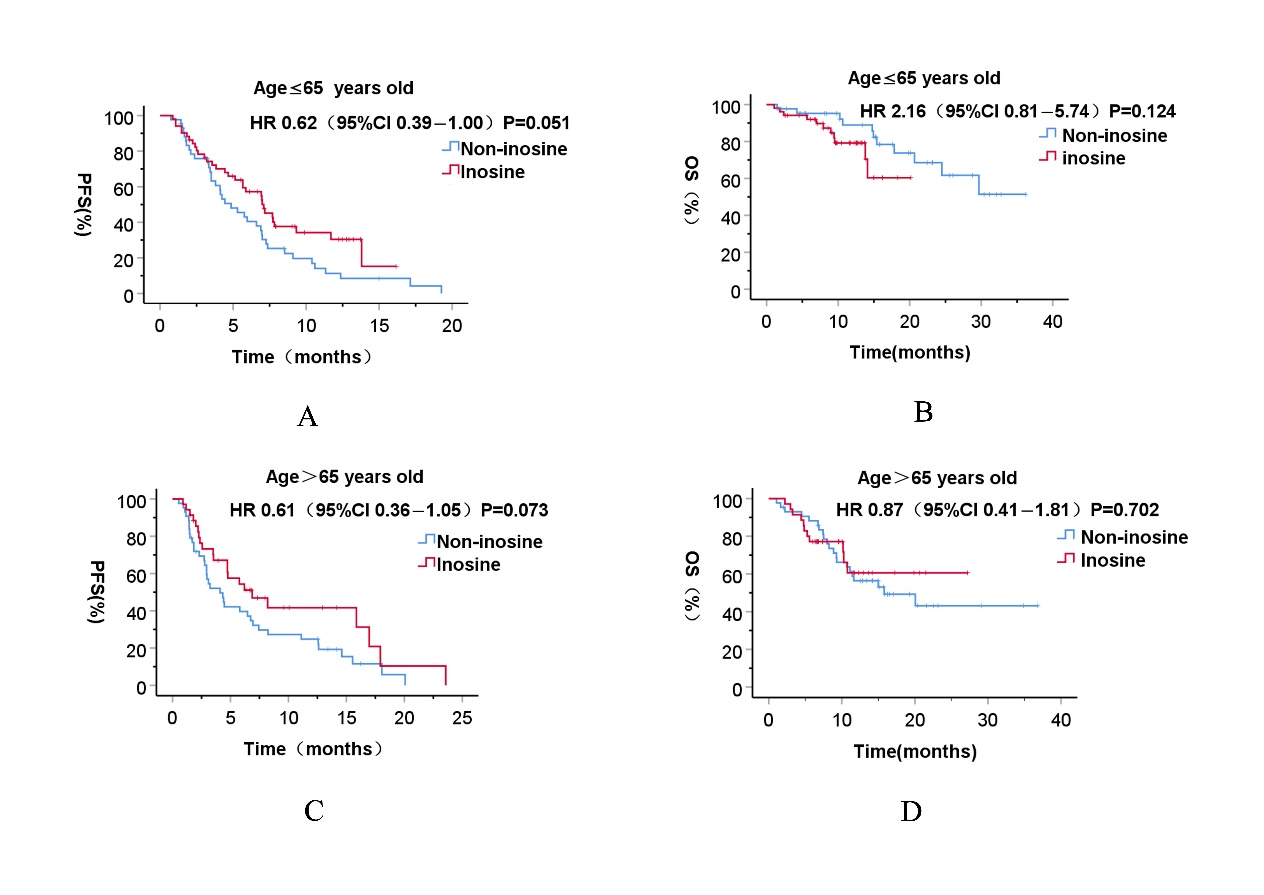


## Figure10. Progression-free survival (PFS) in patients of the age≤65 years old (A) and patients of the age＞65 years old (C),overall survival (OS) in patients of the age≤65 years old (B) and patients of the age＞65 years old (D).95% CI=95% confidence interval, HR=hazard ratio.


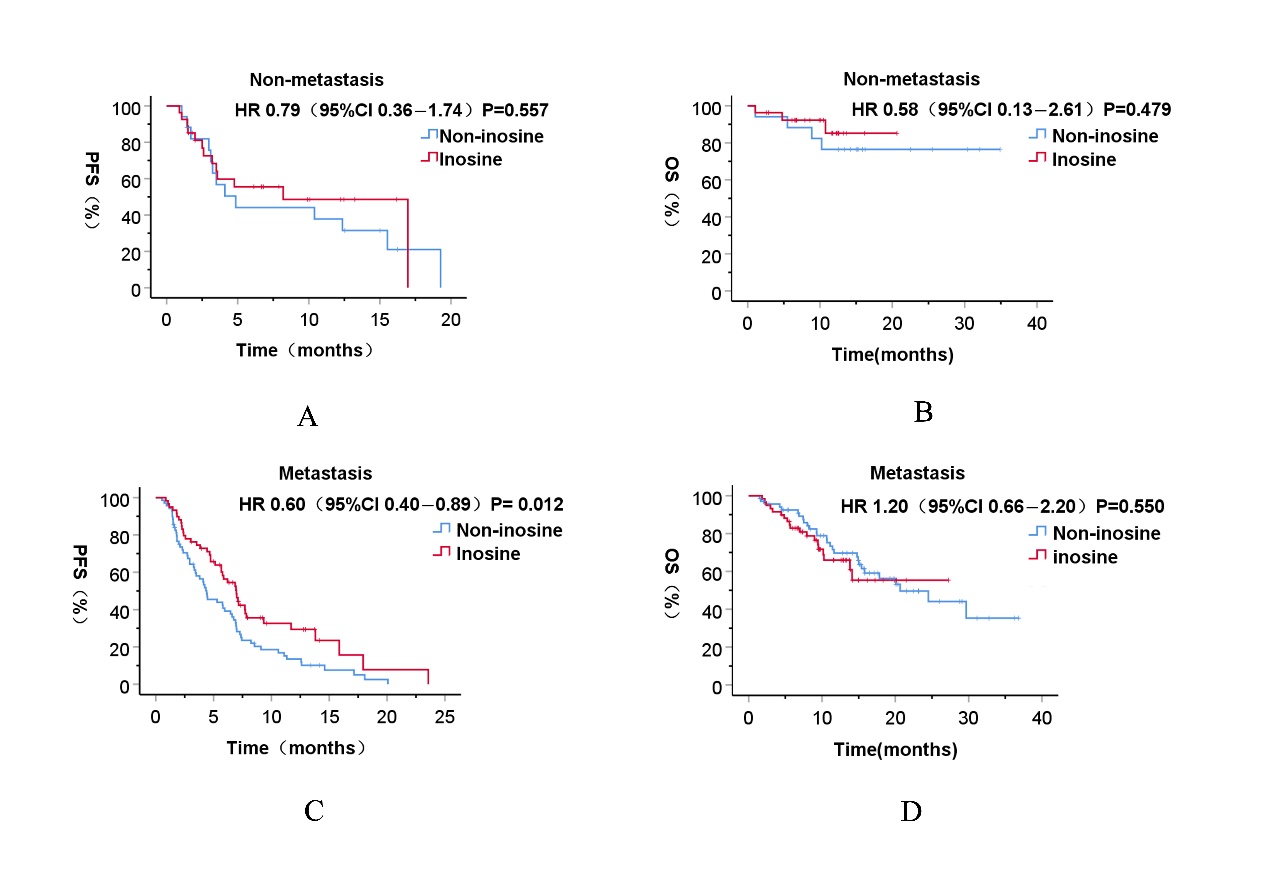


## Figure11.Progression-free survival (PFS) in patients of the no metastasis (A) and patients of the metastasis (C), overall survival (OS) in patients of the no metastasis (B) and patients of the metastasis (D). 95% CI=95% confidence interval, HR=hazard ratio.


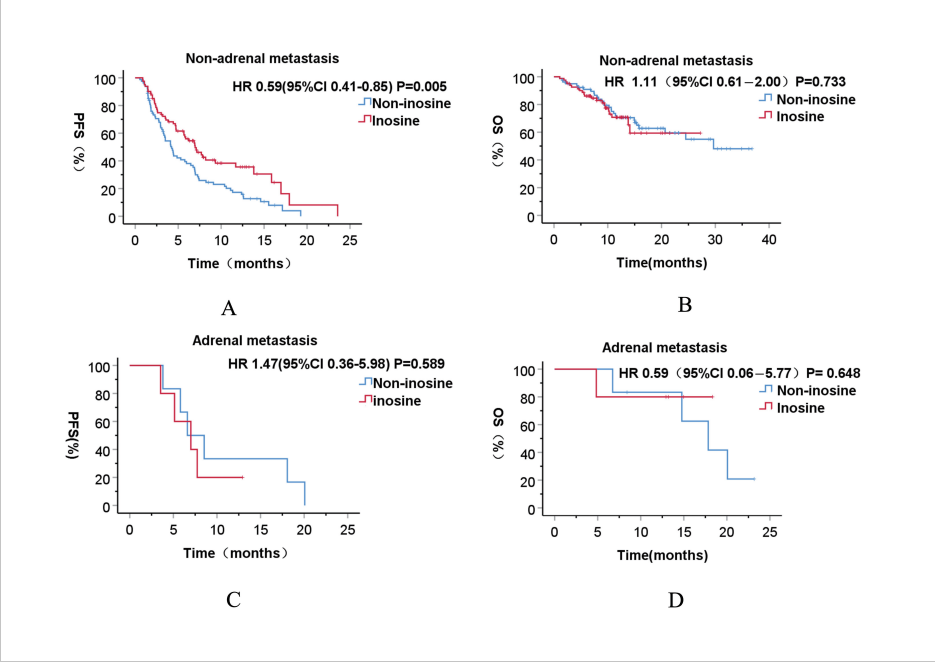


## Figure12. Progression-free survival (PFS) in patients of the non-adrenal metastasis (A) and patients of the adrenal metastasis (C), overall survival (OS) in patients of the non-adrenal metastasis (B) and patients of the adrenal metastasis (D). 95% CI=95% confidence interval, HR=hazard ratio.


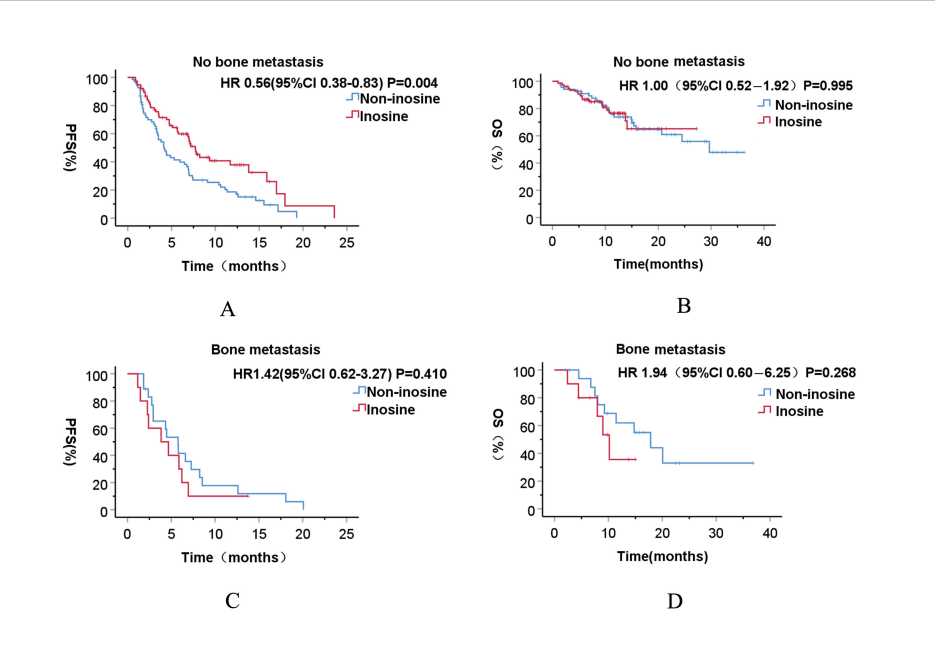


## Figure13. Progression-free survival (PFS) in patients of the no bone metastasis (A) and patients of the bone metastasis (C), overall survival (OS) in patients of the no bone metastasis (B) and patients of the bone metastasis (D).95% CI=95% confidence interval, HR=hazard ratio.


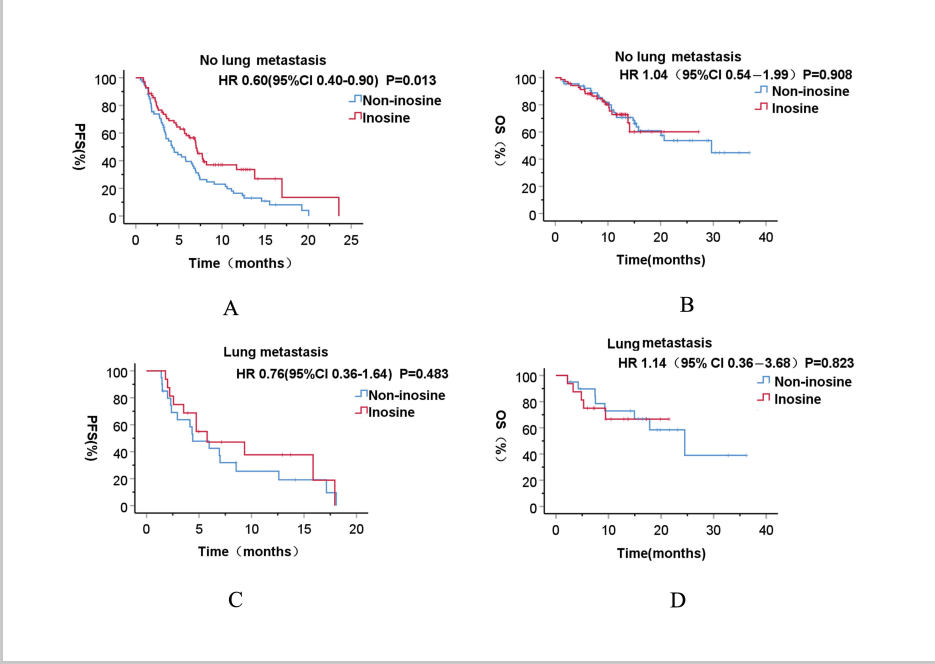


## Figure14.Progression-free survival (PFS) in patients of the no lung metastasis (A) and patients of the lung metastasis (C), overall survival (OS) in patients of the no lung metastasis (B) and patients of the lung metastasis (D). 95% CI=95% confidence interval, HR=hazard ratio.


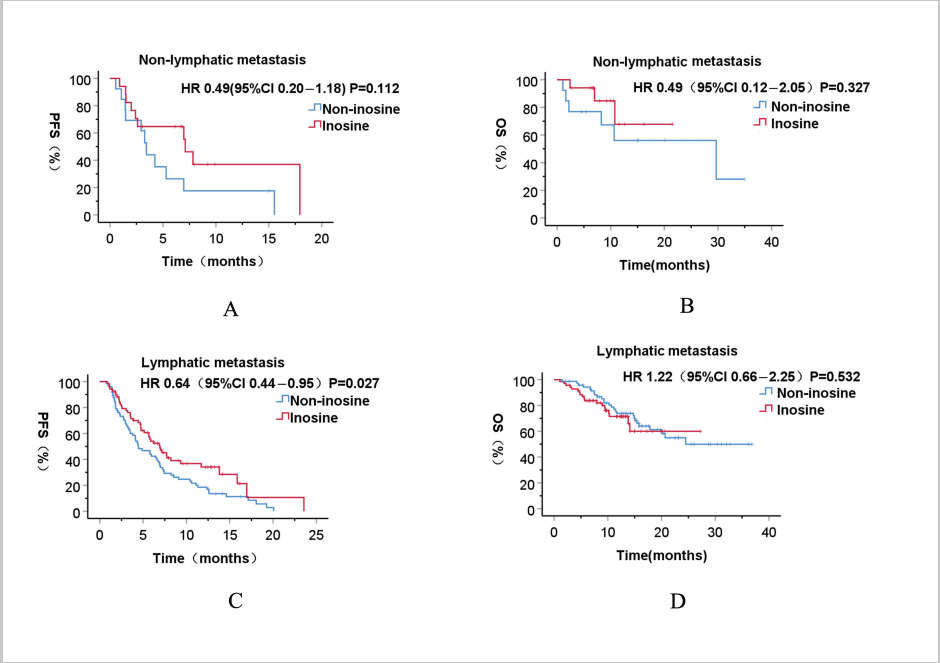


**Figure15**. Progression-free survival (PFS) in patients of the non-lymphatic metastasis (A) and patients of the lymphatic metastasis (C),overall survival (OS) in patients of the non-lymphatic metastasis (B) and patients of the lymphatic metastasis (D). 95%CI=95% confidence interval, HR=hazard ratio.


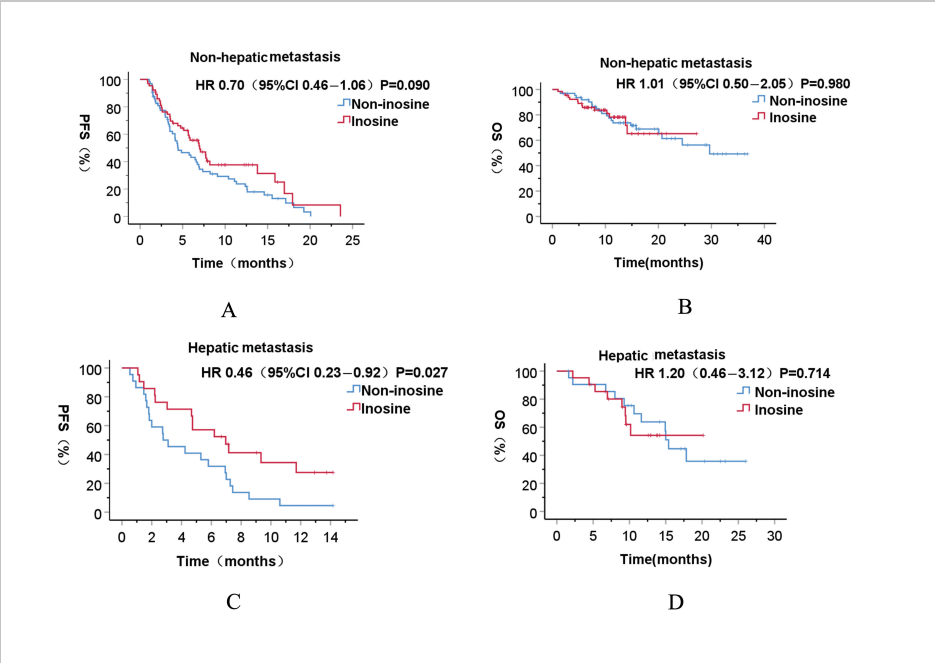


## Figure16.Progression-free survival (PFS) in patients of the Non-hepatic metastasis (A) and patients of the hepatic metastasis (C), overall survival (OS) in patients of the Non-hepatic (B) metastasis and patients of the hepatic metastasis (D). 95% CI=95% confidence interval, HR=hazard ratio.


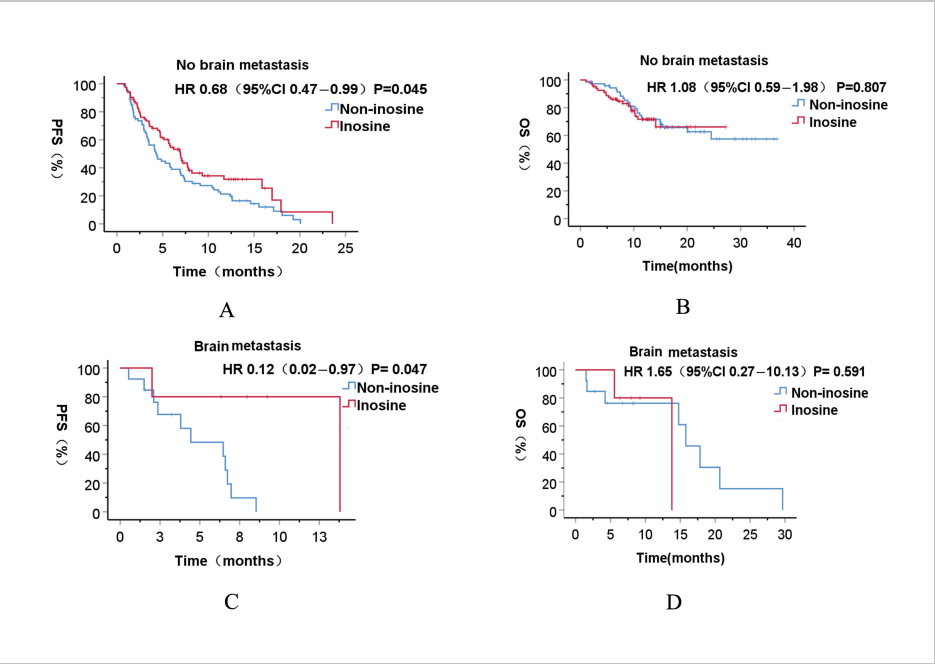


## Figure17.Progression-free survival (PFS) in patients of the no brain metastasis (A) and patients of the brain metastasis (C), overall survival (OS) in patients of the no brain metastasis (B) and patients of the brain metastasis (D). 95% CI=95% confidence interval, HR=hazard ratio.


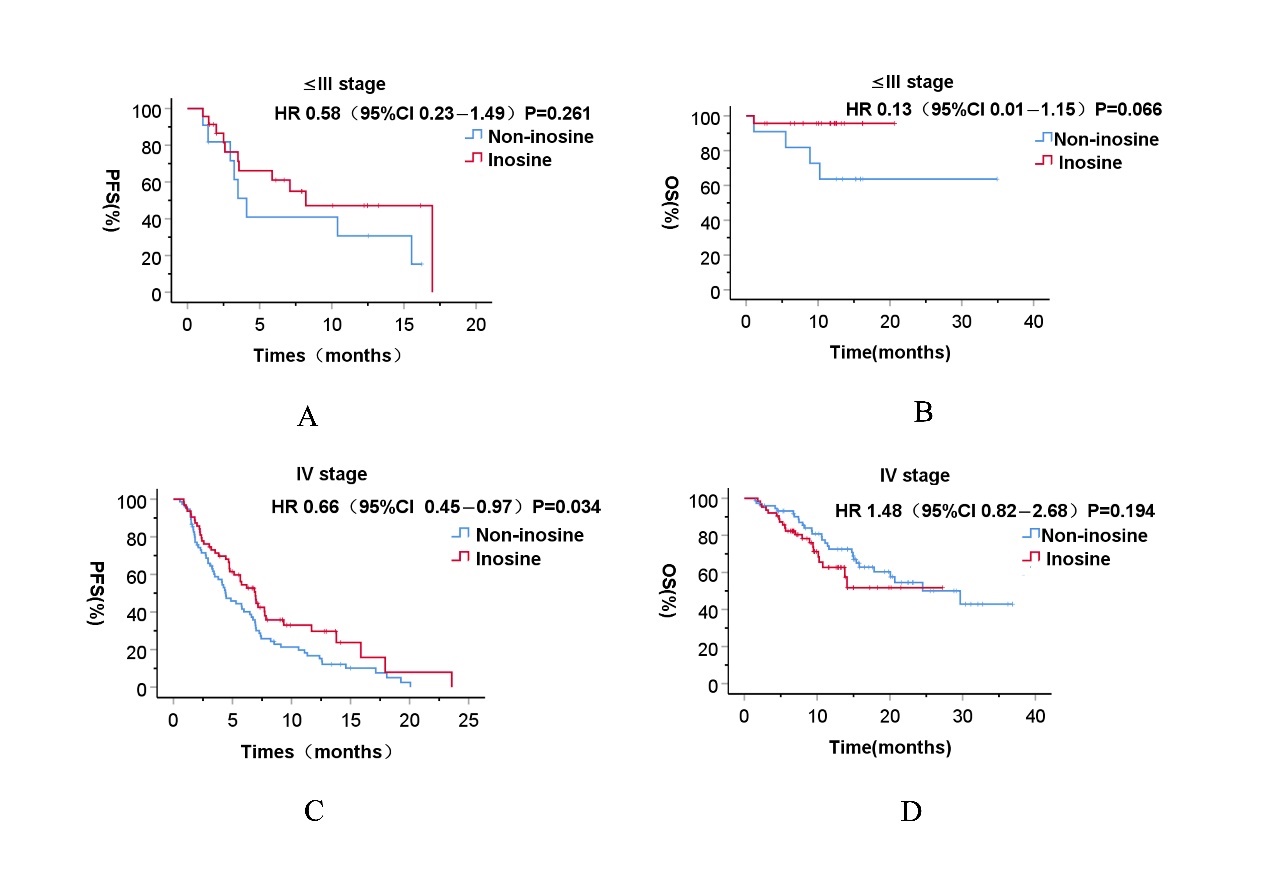


## Figure18.Progression-free survival (PFS) in patients of the ≤Ⅲ stage (A) and patients of the Ⅳ stage (C), overall survival (OS) in patients of the ≤Ⅲ stage (B) and patients of the Ⅳ stage (D). 95% CI=95% confidence interval, HR=hazard ratio.


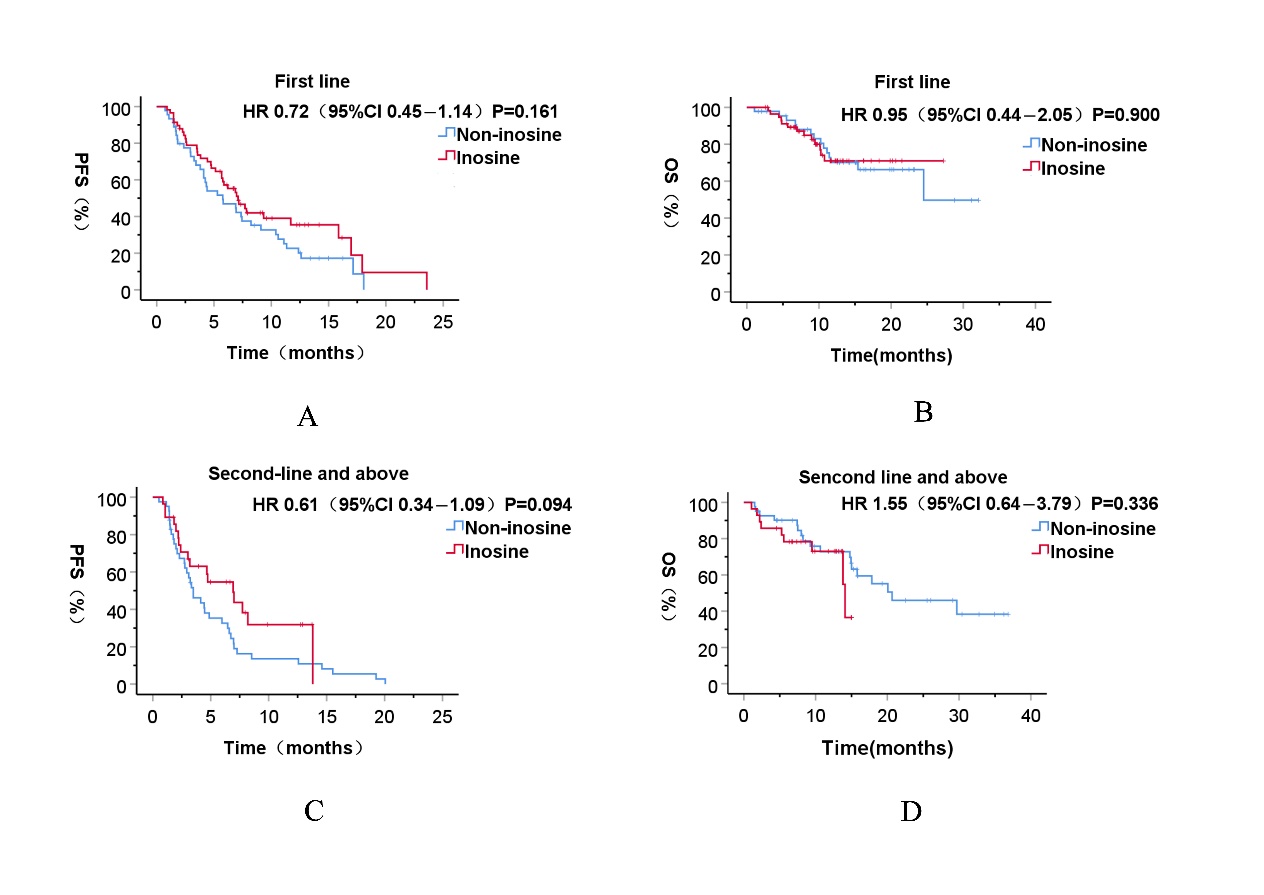


## Figure19.Progression-free survival (PFS) in patients of the first line (A) and patients of the second-line and above (C), overall survival (OS) in patients of the first line (B)and patients of the second-line and above (D). 95% CI=95% confidence interval, HR=hazard ratio.
