# Supplementary material for: Inosine enhances the efficacy of immune‐checkpoint inhibitors in advanced solid tumors: A randomized, controlled, Phase 2 study
Source: Cancer Med. 2024 Sep 13;13(17):e70143. doi: 10.1002/cam4.70143 (PMC11393481; doi:10.1002/cam4.70143)
Supplement: Supplementary file 2 — Appendix S2: [file CAM4-13-e70143-s001.docx]

# Appendix 2-tables

**Inosine Enhances the Efficacy of Immune-checkpoint Inhibitors in Advanced Solid Tumors: A Randomized, Controlled, Phase 2 Study**

Haiqing Zhao, Wei Zhang, Yuting Lu, Yin Dong, Zhihao He, Hongchao Zhen, Qin Li*

**Summary**: The median progression free survival (PFS) was significantly prolonged in the inosine group compared to the control group, and there was a tendency for an increase in the objective response rate (ORR). Inosine had a tendency to enhance the efficacy of ICIs and reduced immunotherapy-related adverse reactions in clinical applications.

## Table 1 ORR of subgroups with statistically different

|  | **Inosine(n/N) (%)** | **Non-inosine(n/N) (%)** | ***P*** |
| --- | --- | --- | --- |
| **Non-drinking** |  |  |  |
| ORR | 22 /66(33.3%) | 6 /57(10.5%) | 0.002 |
| **Non-smoking** |  |  |  |
| ORR | 15/49 (30.6%) | 4 /42(9.5%) | 0.016 |
| **Age >65 years old** |  |  |  |
| ORR | 12/35 (34.3%) | 5 /43(11.6%) | 0.014 |
| **Non-adrenal metastasis** |  |  |  |
| ORR | 21 /81(25.9%) | 10/80 (12.5%) | 0.031 |
| **Non-bone metastasis** |  |  |  |
| ORR | 21/76 (27.6%) | 8 /68(11.8%) | 0.018 |
| **Lung metastasis** |  |  |  |
| ORR | 6 /16(37.5%) | 1 /20(5.0%) | 0.030 |
| **Non-brain metastasis** |  |  |  |
| ORR | 22/81 (27.2%) | 10 /73(13.7%) | 0.040 |

ORR=Objective response rate, 95% CI=95% confidence interval.

## Table 2 Key efficacy indicators in the inosine and the non-inosine groups of the lung cancer

|  | | **Inosine group** (N=31) | **Non-inosine group** (N=26) | *P* |
| --- | --- | --- | --- | --- |
| **ORR n (%)** | | 13(41.9%) | 5(19.2%) | 0.066 |
| **DCR n (%)** | | 29(93.5%) | 22(84.6%) | 0.508 |
| CR n (%) | | 0(%) | 0(%) |  |
| PR n (%) | | 13(41.9%) | 5(19.2%) |  |
| SD n (%) | | 16(51.6%) | 17(65.4%) |  |
| PD n (%) | | 2(6.4%) | 4(15.4%) |  |
| **PFS** | |  |  |  |
| events n (%) | | 17(54.8%) | 21(80.8%) |  |
| mPFS (months) (95% CI) | | 13.8(4.07-23.53) | 4.10(2.28-5.93) | 0.018 |
| 6m-PFS rate (%) | | 67.0% | 39.1% | 0.054 |
| 12m-PFS rate % | | 54.6% | 21.7% | 0.018 |
| **OS** | |  |  |  |
| events n (%) | | 6(19.4%) | 14(53.8%) |  |
| mOS (m) (95% CI) | | NR | 15.80(7.85-23.76) | 0.100 |
| 6m-OS rate (%) | | 90.3% | 92.3% | 1.000 |
| 12m-OS rate (%) | 86.4% | | 58.2% | 0.026 |

ORR=objective response rate, DCR=disease control rate, CR=complete response, PR=partial response, SD=stable disease, PD=progression disease, PFS=progression free survival, mPFS=median progression free survival, 6m-PFS rate=6 months progression free survival, 12m-PFS rate=12 months progression free survival, OS=overall survival, mOS=median overall survival, m=months, 6m-OS rate=6 months overall survival, 12m-OS rate=12 months overall survival, NR=not reaching, 95% CI=95% confidence interval.

## Table 3 Key efficacy indicators in the inosine and the non-inosine groups of the digestive system malignancy

|  | **Inosine**(N=27) | **Non-inosine**(N=28) | *P* |
| --- | --- | --- | --- |
| **ORR n (%)** | 5(18.5%) | 3(10.7%) | 0.661 |
| **DCR n (%)** | 24(88.9%) | 20(71.4%) | 0.106 |
| CR n (%) | 0(0%) | 0(0%) |  |
| PR n (%) | 5(18.5%) | 3(10.7%) |  |
| SD n (%) | 19(70.3%) | 17(60.7%) |  |
| PD n (%) | 3(11.1%) | 8(28.6%) |  |
| **PFS** |  |  |  |
| events n (%) | 22(81.5%) | 26(92.9%) |  |
| mPFS (m) (95% CI) | 5.77 | 3.50 | 0.121 |
| 6m-PFS rate (%) | 49.7% | 27.4% | 0.097 |
| 12m-PFS rate (%) | 7.3% | 3.9% | 0.430 |
| **OS** |  |  |  |
| events n (%) | 8(29.6%) | 10(35.7%) |  |
| mOS (months) (95% CI) | NR | 24.50(11.63-37.37) | 0.819 |
| 6m-OS rate (%) | 79.5% | 88.2% | 0.661 |
| 12m-OS rate (%) | 61.5% | 70.0% | 0.700 |

ORR=objective response rate, DCR=disease control rate, CR=complete response, PR=partial response, SD=stable disease, PD=progression disease, PFS=progression free survival, mPFS=median progression free survival, 6m-PFS rate=6 months progression free survival, 12m-PFS rate=12 months progression free survival, OS=overall survival, mOS=median overall survival, m=months, 6m-OS rate=6 months overall survival, 12m-OS rate=12 months overall survival, NR=not reaching, 95% CI=95% confidence interval.

## Table 4 Key efficacy indicators in the inosine and the non-inosine groups of the PLR<229 and the PLR≥229

|  | | **PLR<229** | | | **PLR≥229** | | |
| --- | --- | --- | --- | --- | --- | --- | --- |
|  | | **Inosine**  (N=64) | **Non-inosine**  (N=58) | ***P*** | **Inosine**  (N=22) | **Non-inosine**  (N=28) | ***P*** |
| **ORR**  n (%) | 18  (28.1%) | 9  (15.5%) | 0.094 | 5  (22.7%) | 4  (14.3%) | 0.689 |  |
| **DCR**  n (%) | 57  (89.1%) | 50  (86.2%) | 0.631 | 19  (86.4%) | 20  (71.4%) | 0.357 |  |
| CR  n (%) | 0  (0%) | 0  (0%) |  | 0  (0%) | 0  (0%) |  |  |
| PR  n (%) | 18  (28.1%) | 9  (15.5%) |  | 5  (22.7%) | 4  (14.3%) |  |  |
| SD  n (%) | 39  (60.9%) | 41  (70.7%) |  | 14  (63.6%) | 16  (57.1%) |  |  |
| PD  n (%) | 7  (10.9%) | 8  (13.8%) |  | 3  (13.6%) | 8  (28.6%) |  |  |
| **PFS** |  |  |  |  |  |  |  |
| events  n (%) | 39(60.9%) | 50  (86.2%) |  | 15(68.1%) | 25  (89.2%) |  |  |
| mPFS (m)  (95% CI) | 7.10  (5.89-8.31) | 4.47  (2.76-6.18) | 0.040 | 6.20  (3.76-8.64) | 3.50  (1.37-5.63) | 0.194 |  |
| 6-mPFS rate  (%) | 57.4% | 42.4% | 0.108 | 52.3% | 35.6% | 0.283 |  |
| 12-mPFS rate  (%) | 36.6% | 22.1% | 0.025 | 27.5% | 9.9% | 0.139 |  |
| **OS** |  |  |  |  |  |  |  |
| events  n (%) | 14(21.9%) | 21  (36.2%) |  | 8(36.4%) | 11  (39.3%) |  |  |
| mOS (m)  (95% CI) | NR | NR |  | 13.80 | 20.07  (2.69-37.44) | 0.577 |  |
| 6-mOS rate  (%) | 85.6% | 92.9% | 0.200 | 85.9% | 88.5% | 1.000 |  |
| 12-mOS rate  (%) | 76.3% | 75.7% | 0.777 | 58.2% | 60.2% | 0.981 |  |

PLR=platelet to lymphocyte ratio, ORR=objective response rate, DCR=disease control rate, CR=complete response, PR=partial response, SD=stable disease, PD=progression disease, PFS=progression free survival, mPFS=median progression free survival, 6m-PFS rate=6 months progression free survival, 12m-PFS rate=12 months progression free survival, OS=overall survival, mOS=median overall survival, m=months, 6m-OS rate=6 months overall survival, 12m-OS rate=12 months overall survival, NR=not reaching, 95% CI=95% confidence interval.

## Table 5 Key efficacy indicators in the inosine and the non-inosine groups of the NLR<4 and the NLR≥4

|  | **NLR<4** | | | **NLR≥4** | | |
| --- | --- | --- | --- | --- | --- | --- |
|  | **Inosine**  (N=53) | **Non-inosine**  (N=61) | ***P*** | **Inosine**  (N=33) | **Non-inosine**  (N=25) | ***P*** |
| **ORR**  n (%) | 14  (26.4%) | 9  (14.8%) | 0.122 | 9  (27.3%) | 4  (16.0%) | 0.308 |
| **DCR**  n (%) | 49  (92.5%) | 49  (80.3%) | 0.063 | 27(81.8%) | 21  (84.0%) | 1.000 |
| CR  n (%) | 0  (0%) | 0  (0%) |  | 0  (0%) | 0  (0%) |  |
| PR  n (%) | 14(26.4%) | 9  (14.8%) |  | 9  (27.3%) | 4  (16.0%) |  |
| SD  n (%) | 35  (66.0%) | 40  (65.6%) |  | 18(54.5%) | 17  (68.0%) |  |
| PD  n (%) | 4  (7.5%) | 12  (19.7%) |  | 6  (18.1%) | 4  (16.0%) |  |
| **PFS** |  |  |  |  |  |  |
| events  n (%) | 29  (54.7%) | 53  (86.9%) |  | 25  (75.8%) | 22  (88.0%) |  |
| mPFS (m)  (95% CI) | 7.10  (6.11-8.09) | 4.33  (2.89-5.77) | 0.022 | 5.87  (3.53-8.38) | 4.43  (1.64-7.22) | 0.272 |
| 6-mPFS rate  (%) | 61.5% | 38.4% | 0.034 | 48.5% | 44.6% | 0.971 |
| 12-mPFS rate  (%) | 35.9% | 20.9% | 0.007 | 31.4% | 10.7% | 0.261 |
| **OS** |  |  |  |  |  |  |
| events  n (%) | 9  (17.0%) | 24  (39.3%) |  | 13(39.4%) | 8  (32.0%) |  |
| mOS (m)  (95% CI) | NR | 20.67  (11.60-29.73) | 0.301 | 14.10  (NR) | 20.67  (10.26-31.08) | 0.209 |
| 6-mOS rate  (%) | 84.2% | 90.0% | 0.394 | 87.9% | 95.7% | 0.536 |
| 12-mOS rate  (%) | 80.7% | 70.4% | 0.167 | 61.0% | 72.9% | 0.261 |

NLR=neutrophil to lymphocyte ratio, ORR=objective response rate, DCR=disease control rate, CR=complete response, PR=partial response, SD=stable disease, PD=progression disease, PFS=progression free survival, mPFS=median progression free survival, 6m-PFS rate=6 months progression free survival, 12m-PFS rate=12 months progression free survival, OS=overall survival, mOS=median overall survival, m=months, 6m-OS rate=6 months overall survival, 12m-OS rate=12 months overall survival, NR=not reaching, 95% CI=95% confidence interval.

## Table 6 Key efficacy indicators in the inosine and the non-inosine groups of the ENR≤0.012 and the ENR>0.012

|  | **ENR≤0.012** | | | **ENR>0.012** | | |
| --- | --- | --- | --- | --- | --- | --- |
|  | **Inosine**  (N=14) | **Non-inosine**  (N=16) | ***P*** | **Inosine**  (N=72) | **Non-inosine**  (N=70) | ***P*** |
| **ORR**  n (%) | 5  (35.7%) | 4  (25.0%) | 0.811 | 18  (25.0%) | 9  (12.9%) | 0.065 |
| **DCR**  n (%) | 12  (85.7%) | 12  (75.0%) | 0.784 | 64  (88.9%) | 58  (82.9%) | 0.302 |
| CR  n (%) | 0  (0%) | 0  (0%) |  | 0  (0%) | 0  (0%) |  |
| PR  n (%) | 5  (35.7%) | 4  (25.0%) |  | 18  (25.0%) | 9  (12.9%) |  |
| SD  n (%) | 7  (50.0%) | 8  (50.0%) |  | 46  (63.9%) | 49  (70.0%) |  |
| PD  n (%) | 2  (14.3%) | 4  (25.0%) |  | 8  (11.1%) | 12  (17.1%) |  |
| **PFS** |  |  |  |  |  |  |
| events  n (%) | 10  (71.4%) | 15  (93.8%) |  | 44  (61.1%) | 60  (85.7%) |  |
| mPFS (m)  (95% CI) | 5.88  (5.55-6.19) | 3.33  (2.86-  3.81) | 0.026 | 7.10  (6.20-8.00) | 5.30  (3.47-7.13) | 0.054 |
| 6-mPFS rate  (%) | 43.8% | 21.1% | 0.257 | 58.0% | 44.5% | 0.133 |
| 12-mPFS rate  (%) | 17.5% | 0% | 0.157 | 37.4% | 22.6% | 0.021 |
| **OS** |  |  |  |  |  |  |
| events  n (%) | 5  (35.7%) | 8  (50.0%) |  | 17  (23.6%) | 24  (34.3%) |  |
| mOS (m)  (95% CI) | NR | 20.67  (14.52-26.82) | 0.973 | NR | NR |  |
| 6-mOS rate  (%) | 85.1% | 93.3% | 0.586 | 85.8% | 91.1% | 0.316 |
| 12-mOS rate  (%) | 58.4% | 71.1% | 0.694 | 75.0% | 71.7% | 0.491 |

ENR=eosinophil-to-neutrophil ratio, ORR=objective response rate, DCR=disease control rate, CR=complete response, PR=partial response, SD=stable disease, PD=progression disease, PFS=progression free survival, mPFS=median progression free survival, 6m-PFS rate=6 months progression free survival, 12m-PFS rate=12 months progression free survival, OS=overall survival, mOS=median overall survival, m=months, 6m-OS rate=6 months overall survival, 12m-OS rate=12 months overall survival, NR=not reaching, 95% CI=95% confidence interval.

## Table 7 Key efficacy indicators in the inosine and the non-inosine groups of the CRP≤8 and the CRP>8

|  | **CRP≤8** | | | **CRP>8** | | |
| --- | --- | --- | --- | --- | --- | --- |
|  | **Inosine**  (N=46) | **Non-inosine**  (N=42) | ***P*** | **Inosine**  (N=40) | **Non-inosine**  (N=44) | ***P*** |
| **ORR**  n (%) | 12  (26.1%) | 10  (23.8%) | 0.805 | 11  (27.5%) | 3  (6.8%) | 0.011 |
| **DCR**  n (%) | 42  (91.3%) | 36  (85.7%) | 0.625 | 34  (85.0%) | 34  (77.3%) | 0.368 |
| CR  n (%) | 0  (0%) | 0  (0%) |  | 0  (0%) | 0  (0%) |  |
| PR  n (%) | 12  (26.1%) | 10  (23.8%) |  | 11  (27.5%) | 3  (6.8%) |  |
| SD  n (%) | 30  (65.2%) | 26  (61.9%) |  | 23  (57.5%) | 31  (70.5%) |  |
| PD  n (%) | 4  (8.7%) | 6  (14.2%) |  | 6  (15.0%) | 10  (22.7%) |  |
| **PFS** |  |  |  |  |  |  |
| events  n (%) | 27  (58.7%) | 34  (81.0%) |  | 27  (67.5%) | 41  (93.2%) |  |
| mPFS (m)  (95% CI) | 7.17  (6.18- 8.15) | 5.30  (3.30-7.31) | 0.129 | 5.87  (3.49-8.24) | 4.10  (2.56-5.64) | 0.052 |
| 6-mPFS rate  (%) | 61.6% | 44.3% | 0.146 | 49.6% | 36.2% | 0.202 |
| 12-mPFS rate  (%) | 42.2% | 22.9% | 0.064 | 24.8% | 14.5% | 0.036 |
| **OS** |  |  |  |  |  |  |
| events  n (%) | 7  (15.2%) | 16  (38.1%) |  | 15  (37.5%) | 16  (36.4%) |  |
| mOS (m)  (95% CI) | NR | 24.50 | 0.324 | NR | 29.67  (13.70-45.6) | 0.188 |
| 6-mOS rate  (%) | 93.4% | 92.5% | 1.000 | 76.6% | 90.6% | 0.090 |
| 12-mOS rate  (%) | 86.6% | 70.9% | 0.063 | 51.7% | 71.8% | 0.216 |

CRP=C-Reactive protein, ORR=objective response rate, DCR=disease control rate, CR=complete response, PR=partial response, SD=stable disease, PD=progression disease, PFS=progression free survival, mPFS=median progression free survival, 6m-PFS rate=6 months progression free survival, 12m-PFS rate=12 months progression free survival, OS=overall survival, mOS=median overall survival, m=months, 6m-OS rate=6 months overall survival, 12m-OS rate=12 months overall survival, NR=not reaching, 95% CI=95% confidence interval.

## Table 8 Key efficacy indicators in the inosine and the non-inosine groups of the LDH<216 and the LDH≥216

|  | **LDH<216** | | | **LDH≥216** | | |
| --- | --- | --- | --- | --- | --- | --- |
|  | **Inosine**  (N=59) | **Non-inosine**  (N=57) | ***P*** | **Inosine**  (N=27) | **Non-inosine**  (N=29) | ***P*** |
| **ORR**  n (%) | 19  (32.2%) | 9  (15.8%) | 0.039 | 4  (14.8%) | 4  (13.8%) | 1.000 |
| **DCR**  n (%) | 53  (89.8) | 48  (84.2%) | 0.367 | 23  (85.2%) | 22  (75.9%) | 0.380 |
| CR  n (%) | 0  (0%) | 0  (0%) |  | 0  (0%) | 0  (0%) |  |
| PR  n (%) | 19  (32.2%) | 9  (15.8%) |  | 4  (14.8) | 4  (13.8%) |  |
| SD  n (%) | 34  (57.6%) | 39  (68.4%) |  | 19  (18.1%) | 18  (62.1%) |  |
| PD  n (%) | 6  (10.2%) | 9  (15.8%) |  | 4  (14.8%) | 7  (24.1%) |  |
| **PFS** |  |  |  |  |  |  |
| events  n (%) | 37  (62.7%) | 47  (82.4%) |  | 17  (63.0%) | 28  (96.6%) |  |
| mPFS (m)  (95%CI) | 7.00  (5.53-8.47) | 4.47  (2.65-6.28) | 0.291 | 7.10  (3.78-10.42) | 2.93  (1.23-4.63) | 0.010 |
| 6-mPFS rate  (%) | 59.1% | 43.0% | 0.140 | 50.0% | 34.5% | 0.189 |
| 12-mPFS rate  (%) | 31.6% | 20.8% | 0.107 | 40.4% | 13.8% | 0.011 |
| **OS** |  |  |  |  |  |  |
| events  n (%) | 13  (22.0%) | 14  (24.6%) |  | 9  (33.3%) | 18  (62.1%) |  |
| mOS (m)  (95% CI) | NR | NR |  | 14.77  (9.65-19.89) | NR | 0.290 |
| 6-mOS rate  (%) | 87.8% | 96.4% | 0.182 | 81.0% | 82.5% | 1.000 |
| 12-mOS rate  (%) | 74.1% | 82.0% | 0.525 | 67.4% | 51.4% | 0.240 |

LDH=lactate dehydrogenase, ORR=objective response rate, DCR=disease control rate, CR=complete response, PR=partial response, SD=stable disease, PD=progression disease, PFS=progression free survival, mPFS=median progression free survival, 6m-PFS rate=6 months progression free survival, 12m-PFS rate=12 months progression free survival, OS=overall survival, mOS=median overall survival, m=months, 6m-OS rate=6 months overall survival, 12m-OS rate=12 months overall survival, NR=not reaching, 95% CI=95% confidence interval.

## Table 9 Key efficacy indicators in the inosine and the non-inosine groups of the non-drinking and drinking

|  | **Non-drinking** | | | **Drinking** | | |
| --- | --- | --- | --- | --- | --- | --- |
|  | **Inosine**  (N=66) | **Non-inosine**  (N=57) | ***P*** | **Inosine**  (N=20) | **Non-inosine**  (N=29) | ***P*** |
| **ORR**  n (%) | 22(33.3%) | 6  (10.5%) | 0.002 | 1  (5.0%) | 7  (24.1%) | 0.165 |
| **DCR**  n (%) | 60(90.9%) | 44  (77.2%) | 0.036 | 16(80.0%) | 26(89.7%) | 0.593 |
| CR  n (%) | 0(0%) | 0(0%) |  | 0(0%) | 0(0%) |  |
| PR  n (%) | 22  (33.3%) | 6  (10.5%) |  | 1  (5.0%) | 7  (24.1%) |  |
| SD  n (%) | 38  (57.6%) | 38  (66.7%) |  | 15  (75.0%) | 19(65.5%) |  |
| PD  n (%) | 6  (9.0%) | 13  (22.8%) |  | 4  (20.0%) | 3  (10.3%) |  |
| **PFS** |  |  |  |  |  |  |
| events  n (%) | 42  (63.6%) | 51  (89.4%) |  | 12  (60.0%) | 24  (82.8%) |  |
| mPFS (m)  (95% CI) | 7.00  (5.93-8.05) | 4.43  (2.72-6.15) | 0.029 | 8.20  (2.43-13.97) | 4.10  (2.83-5.37) | 0.239 |
| 6-mPFS rate  (%) | 57.7% | 40.3% | 0.060 | 50.7% | 40.1% | 0.296 |
| 12-mPFS rate  (%) | 34.8% | 21.6% | 0.037 | 33.8% | 12.0% | 0.070 |
| **OS** |  |  |  |  |  |  |
| events  n (%) | 19  (28.8%) | 19  (33.3%) |  | 3  (15.0%) | 13  (44.8%) |  |
| mOS (m)  (95% CI) | NR | NR |  | NR | 15.80  (8.10-23.50) | 0.288 |
| 6-mOS rate  (%) | 84.8% | 89.1% | 0.447 | 89.4% | 96.6% | 0.738 |
| 12-mOS rate  (%) | 69.5% | 77.1% | 0.540 | 81.3% | 58.8% | 0.129 |

ORR=objective response rate, DCR=disease control rate, CR=complete response, PR=partial response, SD=stable disease, PD=progression disease, PFS=progression free survival, mPFS=median progression free survival, 6m-PFS rate=6 months progression free survival, 12m-PFS rate=12 months progression free survival, OS=overall survival, mOS=median overall survival, m=months, 6m-OS rate=6 months overall survival, 12m-OS rate=12 months overall survival, NR=not reaching, 95% CI=95% confidence interval.

## Table 10 Key efficacy indicators in the inosine and the non-inosine groups of the smoking and the non-smoking group

|  | **Non-smoking** | | | **smoking** | | |
| --- | --- | --- | --- | --- | --- | --- |
|  | **Inosine**  (N=49) | **Non-inosine**  (N=42) | ***P*** | **Inosine**  (N=37) | **Non-inosine**  (N=44) | ***P*** |
| **ORR**  n (%) | 15  (30.6%) | 4  (9.5%) | 0.014 | 8  (21.6%) | 9  (20.5%) | 0.898 |
| **DCR**  n (%) | 43  (87.8%) | 32  (76.2%) | 0.149 | 33  (89.2%) | 38  (86.4%) | 0.963 |
| CR  n (%) | 0  (0%) | 0  (0%) |  | 0  (0%) | 0  (0%) |  |
| PR  n (%) | 15  (30.6%) | 4  (9.5%) |  | 8  (21.6%) | 9  (20.5%) |  |
| SD  n (%) | 28  (57.1%) | 28  (66.7%) |  | 25  (67.6%) | 29  (65.9%) |  |
| PD  n (%) | 6  (12.2%) | 10  (23.8%) |  | 4  (10.8%) | 6  (13.6%) |  |
| **PFS** |  |  |  |  |  |  |
| events  n (%) | 32  (65.3%) | 37  (88.1%) |  | 22(59.5%) | 38  (86.4%) |  |
| mPFS (m)  (95% CI) | 6.93  (5.41-8.46) | 4.43  (3.53-5.33) | 0.094 | 7.10  (3.41-10.79) | 4.23  (1.43-7.04) | 0.046 |
| 6-mPFS rate  (%) | 55.1% | 40.4% | 0.174 | 57.5% | 40.0% | 0.144 |
| 12-mPFS rate  (%) | 34.1% | 19.6% | 0.085 | 35.7% | 17.5% | 0.027 |
| **OS** |  |  |  |  |  |  |
| events  n (%) | 16  (32.7%) | 14  (33.3%) |  | 6  (16.2%) | 18  (40.9%) |  |
| mOS (m)  (95% CI) | NR | 29.67 | 0.131 | NR | 24.50  (11.76-37.24) | 0.159 |
| 6-mOS rate  (%) | 83.4% | 90.2% | 0.339 | 88.9% | 93.0% | 0.810 |
| 12-mOS rate  (%) | 64.9% | 79.4% | 0.290 | 81.3% | 63.3% | 0.105 |

ORR=objective response rate, DCR=disease control rate, CR=complete response, PR=partial response, SD=stable disease, PD=progression disease, PFS=progression free survival, mPFS=median progression free survival, 6m-PFS rate=6 months progression free survival, 12m-PFS rate=12 months progression free survival, OS=overall survival, mOS=median overall survival, m=months, 6m-OS rate=6 months overall survival, 12m-OS rate=12 months overall survival, NR=not reaching, 95% CI=95% confidence interval.

## Table 11 Key efficacy indicators in the inosine and the non-inosine groups of the ECOG=0 and ECOG≥1

|  | | **ECOG=0** | | | **ECOG≥1** | | |
| --- | --- | --- | --- | --- | --- | --- | --- |
|  | **Inosine**  (N=28) | | **Non-inosine**  (N=31) | ***P*** | **Inosine**  (N=58) | **Non-inosine**  (N=55) | ***P*** |
| **ORR**  n (%) | 12  (42.9%) | | 6  (19.4%) | 0.050 | 11  (19.0%) | 7  (12.7%) | 0.365 |
| **DCR**  n (%) | 25  (89.3%) | | 28  (90.3%) | 1.000 | 51  (87.9%) | 42  (76.4%) | 0.107 |
| CR  n (%) | 0  (0%) | | 0  (0%) |  | 0  (0%) | 0  (0%) |  |
| PR  n (%) | 12  (42.9%) | | 6  (19.4%) |  | 11  (19.0%) | 7  (12.7%) |  |
| SD  n (%) | 13  (46.4%) | | 22  (71.0%) |  | 40  (67.0%) | 35  (63.6%) |  |
| PD  n (%) | 3  (10.7%) | | 3  (9.7%) |  | 7  (12.1%) | 13  (23.6%) |  |
| **PFS** |  | |  |  |  |  |  |
| events  n (%) | 16  (57.1%) | | 26  (83.9%) |  | 38  (65.5%) | 49  (89.1%) |  |
| mPFS (m)  (95% CI) | 8.20  (2.72 -13.68) | | 6.97  (5.60-8.34) | 0.416 | 6.87  (5.52-8.21) | 4.10  (2.78-5.42) | 0.004 |
| 6-mPFS rate  (%) | 59.7% | | 56.9% | 0.836 | 54.3% | 30.2% | 0.017 |
| 12-mPFS rate  (%) | 45.2% | | 25.4% | 0.099 | 28.7% | 14.1% | 0.023 |
| **OS** |  | |  |  |  |  |  |
| events  n (%) | 4  (14.3%) | | 6  (19.4%) |  | 18(31.0%) | 26  (47.3%) |  |
| mOS (m)  (95% CI) | NR | | NR |  | NR | 20.07  (13.93-26.20) | 0.536 |
| 6-mOS rate  (%) | 92.9% | | 100% | 0.427 | 82.6% | 86.8% | 0.502 |
| 12-mOS rate  (%) | 81.6% | | 92.6% | 0.574 | 67.8% | 59.2% | 0.317 |

ECOG=Eastern Cooperative Oncology Group, ORR=objective response rate, DCR=disease control rate, CR=complete response, PR=partial response, SD=stable disease, PD=progression disease, PFS=progression free survival, mPFS=median progression free survival, 6m-PFS rate=6 months progression free survival, 12m-PFS rate=12 months progression free survival, OS=overall survival, mOS=median overall survival, m=months, 6m-OS rate=6 months overall survival, 12m-OS rate=12 months overall survival, NR=not reaching, 95% CI=95% confidence interval.

## Table 12 Key efficacy indicators in the inosine and the non-inosine groups of the female and the male

|  | **Female** | | | **Male** | |  | |
| --- | --- | --- | --- | --- | --- | --- | --- |
|  | **Inosine**  (N=27) | **Non-inosine**  (N=37) | ***P*** | **Inosine**  (N=59) | **Non-inosine**  (N=49) | | ***P*** |
| **ORR** | 5(18.5%) | 4  (10.8%) | 0.609 | 18  (30.5%) | 9  (18.4%) | | 0.147 |
| n (%) |  |  |  |  |  |  |  |
| **DCR** | 23(85.2%) | 29  (78.4%) | 0.491 | 53  (89.8%) | 41  (83.7%) | | 0.343 |
| n (%) |  |  |  |  |  |  |  |
| CR | 0  (0%) | 0  (0%) |  | 0  (0%) | 0  (0%) | |  |
| n (%) |  |  |  |  |  |  |  |
| PR | 5  (18.5%) | 4  (10.8%) |  | 18  (30.5%) | 9  (18.4%) | |  |
| n (%) |  |  |  |  |  |  |  |
| SD | 18  (66.7%) | 25  (67.6%) |  | 35  (59.3%) | 32  (65.3%) | |  |
| n (%) |  |  |  |  |  |  |  |
| PD | 4  (14.8%) | 8  (21.6%) |  | 6  (10.2%) | 8  (16.3%) | |  |
| n (%) |  |  |  |  |  |  |  |
| **PFS** |  |  |  |  |  | |  |
| events  n (%) | 14  (51.9%) | 34  (91.9%) |  | 40  (67.8%) | 41  (83.7%) | |  |
| mPFS (m)  (95% CI) | 7.83  (2.43-13.23) | 4.40  (3.93-4.87) | 0.062 | 6.93  (5.47-8.38) | 4.23  (2.26-6.20) | | 0.135 |
| 6-mPFS rate  (%) | 65.1% | 37.9% | 0.039 | 52.2% | 41.8% | | 0.334 |
| 12-mPFSrate (%) | 38.9% | 17.5% | 0.012 | 32.3% | 19.2% | | 0.109 |
| **OS** |  |  |  |  |  | |  |
| events  n (%) | 8  (29.6%) | 13  (35.1%) |  | 14  (23.7%) | 19  (38.8%) | |  |
| mOS (m)  (95% CI) | 14.10  (10.09-18.12) | NR | 0.306 | NR | 29.67  (10.65-48.68) | | 0.658 |
| 6-mOS rate  (%) | 85.0% | 88.7% | 0.924 | 86.0% | 93.7% | | 0.341 |
| 12-mOS rate  (%) | 71.8% | 75.9% | 0.954 | 71.7% | 68.1% | | 1.000 |

ORR=objective response rate, DCR=disease control rate, CR=complete response, PR=partial response, SD=stable disease, PD=progression disease, PFS=progression free survival, mPFS=median progression free survival, 6m-PFS rate=6 months progression free survival, 12m-PFS rate=12 months progression free survival, OS=overall survival, mOS=median overall survival, m=months, 6m-OS rate=6 months overall survival, 12m-OS rate=12 months overall survival, NR=not reaching, 95% CI=95% confidence interval.

## Table 13 Key efficacy indicators in the inosine and the non-inosine groups of the age≤65 years old and age>65 years old

|  | **Age≤65 years old** | | | **Age>65 years old** | | |
| --- | --- | --- | --- | --- | --- | --- |
|  | **Inosine**  (N=51) | **Non-inosine**  (N=43) | ***P*** | **Inosine**  (N=35) | **Non-inosine**  (N=43) | ***P*** |
| **ORR**  n (%) | 11  (21.6%) | 8  (18.6%) | 0.721 | 12  (34.3%) | 5  (11.6%) | 0.016 |
| **DCR**  n (%) | 44  (86.3%) | 36  (83.7%) | 0.729 | 32  (91.4%) | 34  (79.1%) | 0.132 |
| CR  n (%) | 0  (0%) | 0  (0%) |  | 0  (0%) | 0  (0%) |  |
| PR  n (%) | 11  (21.6%) | 8  (18.6%) |  | 12  (34.3%) | 5  (11.6%) |  |
| SD  n (%) | 33  (64.7%) | 28  (65.1%) |  | 20  (57.1%) | 29  (67.4%) |  |
| PD  n (%) | 7  (13.7%) | 7  (16.3%) |  | 3  (8.6%) | 9  (20.9%) |  |
| **PFS** |  |  |  |  |  |  |
| events  n (%) | 32(62.7%) | 38  (88.4%) |  | 22  (62.9%) | 37  (86.0%) |  |
| mPFS (m)  (95% CI) | 7.10  (5.28-8.92) | 4.8  (2.87-6.87) | 0.051 | 6.87  (3.03-10.70) | 4.10  (2.33-5.87) | 0.073 |
| 6-mPFS rate  n (%) | 57.2% | 40.4% | 0.157 | 54.3% | 39.7% | 0.179 |
| 12-mPFS rate  n (%) | 30.4% | 11.2% | 0.029 | 41.7% | 24.8% | 0.060 |
| **OS** |  |  |  |  |  |  |
| events  n (%) | 11(21.6%) | 11  (25.6%) |  | 11  (31.4%) | 21  (48.8%) |  |
| mOS (m)  (95% CI) | NR | NR |  | NR | 15.80  (5.53-26.07) | 0.702 |
| 6-mOS rate  (%) | 92.0% | 95.2% | 0.836 | 77.1% | 88.2% | 0.186 |
| 12-mOS rate  (%) | 79.2% | 88.9% | 0.243 | 60.6% | 56.4% | 0.343 |

ORR=objective response rate, DCR=disease control rate, CR=complete response, PR=partial response, SD=stable disease, PD=progression disease, PFS=progression free survival, mPFS=median progression free survival, 6m-PFS rate=6 months progression free survival, 12m-PFS rate=12 months progression free survival, OS=overall survival, mOS=median overall survival, m=months, 6m-OS rate=6 months overall survival, 12m-OS rate=12 months overall survival, NR=not reaching, 95% CI=95% confidence interval.

## Table 14 Key efficacy indicators in the inosine and the non-inosine groups of the non-metastasis and the metastasis

|  | **Non-metastasis** | | | **Metastasis** | | | |
| --- | --- | --- | --- | --- | --- | --- | --- |
|  | **Inosine**  (N=27) | **Non-inosine**  (N=17) | ***P*** | | **Inosine**  (N=59) | **Non-inosine**  (N=69) | ***P*** |
| **ORR**  n (%) | 8  (29.6%) | 3  (17.6%) | 0.592 | | 15  (25.4%) | 10  (14.5%) | 0.120 |
| **DCR**  n (%) | 24  (88.9%) | 15  (88.2%) | 1.000 | | 52  (88.1%) | 55  (79.7%) | 0.199 |
| CR  n (%) | 0  (0%) | 0  (0%) |  | | 0  (0%) | 0  (0%) |  |
| PR  n (%) | 8  (29.6%) | 3  (17.6%) |  | | 15  (25.4%) | 10  (14.5%) |  |
| SD  n (%) | 16  (59.3%) | 12  (70.6%) |  | | 37  (62.7%) | 45  (65.2%) |  |
| PD  n (%) | 3  (11.1%) | 2  (11.8%) |  | | 7  (11.9%) | 14  (20.3%) |  |
| **PFS** |  |  |  | |  |  |  |
| events  n (%) | 13  (48.1%) | 13  (76.5%) |  | | 41  (69.5%) | 62  (89.9%) |  |
| mPFS (m)  (95% CI) | 8.20  (3.48-12.92) | 4.87  (2.23-7.51) | 0.557 | | 6.97  (5.60-8.33) | 4.40  (2.95-5.85) | 0.012 |
| 6-mPFS rate  (%) | 55.5% | 44.1% | 0.429 | | 56.4% | 39.2% | 0.078 |
| 12-mPFS rate  (%) | 48.6% | 37.8% | 0.353 | | 29.4% | 13.5% | 0.020 |
| **OS** |  |  |  | |  |  |  |
| events  n (%) | 3  (11.1%) | 4  (23.5%) |  | | 19  (32.2%) | 28  (40.6%) |  |
| mOS (m)  (95% CI) | NR | NR |  | | NR | 20.67  (12.53-28.80) | 0.550 |
| 6-mOS rate  (%) | 92.3% | 88.2% | 1.000 | | 82.8% | 92.5% | 0.089 |
| 12-mOS rate  (%) | 85.2% | 76.5% | 0.501 | | 66.0% | 69.7% | 0.730 |

ORR=objective response rate, DCR=disease control rate, CR=complete response, PR=partial response, SD=stable disease, PD=progression disease, PFS=progression free survival, mPFS=median progression free survival, 6m-PFS rate=6 months progression free survival, 12m-PFS rate=12 months progression free survival, OS=overall survival, mOS=median overall survival, m=months, 6m-OS rate=6 months overall survival, 12m-OS rate=12 months overall survival, NR=not reaching, 95% CI=95% confidence interval.

## Table 15 Key efficacy indicators in the inosine and the non-inosine groups of the non-adrenal metastasis and the adrenal metastasis

|  | **Non-adrenal metastasis** | | | **Adrenal metastasis** | | |
| --- | --- | --- | --- | --- | --- | --- |
|  | **Inosine**  (N=81) | **Non-inosine**  (N=80) | ***P*** | **Inosine**  (N=5) | **Non-inosine**  (N=6) | ***P*** |
| **ORR**  n (%) | 21  (25.9%) | 10  (12.5%) | 0.031 | 2  (40.0%) | 3  (50.0%) | 1.000 |
| **DCR**  n (%) | 71  (87.7%) | 64  (80.0%) | 0.187 | 5  (100%) | 6  (100%) |  |
| CR  n (%) | 0  (0%) | 0  (0%) |  | 0  (0%) | 0  (0%) |  |
| PR  n (%) | 21  (25.9%) | 10  (12.5%) |  | 2  (40.0%) | 3  (50.0%) |  |
| SD  n (%) | 50  (61.7%) | 54  (67.5%) |  | 3  (60.0%) | 3  (50.0%) |  |
| PD  n (%) | 10  (12.3%) | 16  (20.0%) |  | 0  (0%) | 0  (0%) |  |
| **PFS** |  |  |  |  |  |  |
| events  n (%) | 50  (61.7%) | 69  (86.3%) |  | 4  (80.0%) | 6  (100%) |  |
| mPFS (m)  (95% CI) | 6.97  (5.14-8.80) | 4.23  (3.31-5.16) | 0.005 | 7.00  (2.99-11.01) | 6.60  (3.32-9.89) | 0.589 |
| 6-mPFS rate  (%) | 55.9% | 38.1% | 0.033 | 60.0% | 66.7% | 1.000 |
| 12-mPFS rate (%) | 35.6% | 17.3% | 0.003 | 20.0% | 33.3% | 1.000 |
| **OS** |  |  |  |  |  |  |
| events  n (%) | 21  (25.9%) | 28  (35.0%) |  | 1  (20.0%) | 4  (66.7%) |  |
| mOS (m)  (95% CI) | NR | NR |  | NR | 17.83  (11.43-24.23) |  |
| 6-mOS rate  (%) | 86.1% | 90.9% | 0.331 | 80.0% | 100% | 1.000 |
| 12-mOS rate (%) | 70.7% | 70.5% | 0.682 | 80.0% | 83.3% | 1.000 |

ORR=objective response rate, DCR=disease control rate, CR=complete response, PR=partial response, SD=stable disease, PD=progression disease, PFS=progression free survival, mPFS=median progression free survival, 6m-PFS rate=6 months progression free survival, 12m-PFS rate=12 months progression free survival, OS=overall survival, mOS=median overall survival, m=months, 6m-OS rate=6 months overall survival, 12m-OS rate=12 months overall survival, NR=not reaching, 95% CI=95% confidence interval.

## Table 16 Key efficacy indicators in the inosine and the non-inosine groups of the no bone metastasis and the bone metastasis

|  | **No bone metastasis group** | | | **Bone metastasis group** | | | |
| --- | --- | --- | --- | --- | --- | --- | --- |
|  | **Inosine**  (N=76) | **Non-inosine**  (N=68) | ***P*** | **Inosine**  (N=10) | **Non-inosine**  (N=18) | | ***P*** |
| **ORR**  n (%) | 21  (27.6%) | 8  (11.8%) | 0.018 | 2  (20.0%) | | 5  (27.8%) | 1.000 |
| **DCR**  n (%) | 68  (89.5%) | 55  (80.9%) | 0.145 | 8  (80.0%) | | 15  (83.3%) | 1.000 |
| CR  n (%) | 0  (0%) | 0  (0%) |  | 0  (0%) | | 0  (0%) |  |
| PR  n (%) | 21  (27.6%) | 8  (11.8%) |  | 2  (20.0%) | | 5  (27.8%) |  |
| SD  n (%) | 47  (61.8%) | 47  (69.1%) |  | 6  (60.0%) | | 10  (55.6%) |  |
| PD  n (%) | 8  (10.5%) | 13  (19.1%) |  | 2  (20.0%) | | 3  (16.7%) |  |
| **PFS** |  |  |  |  | |  |  |
| events  n (%) | 45  (59.2%) | 58  (85.3%) |  | 9  (90.0%) | | 17  (94.4%) |  |
| mPFS (m)  (95% CI) | 7.70  (6.56-8.84) | 4.13  (3.11-5.16) | 0.004 | 3.83  (0.32-  7.35) | | 5.77  (3.81-  7.73) | 0.410 |
| 6-mPFS rate  (%) | 59.8% | 39.8% | 0.018 | 30.0% | | 41.5% | 0.689 |
| 12-mPFS rate  (%) | 37.8% | 18.6% | 0.003 | 10.0% | | 17.8% | 0.629 |
| **OS** |  |  |  |  | |  |  |
| events  n (%) | 17  (22.4%) | 23  (33.8%) |  | 5  (50.0%) | | 9  (50.0%) |  |
| mOS (m)  (95% CI) | NR | NR |  | 10.17  (7.54-12.79) | | 17.83  (10.10-25.56) | 0.268 |
| 6-mOS rate  (%) | 86.5% | 90.9% | 0.409 | 80.0% | | 93.8% | 0.284 |
| 12-mOS rate  (%) | 76.5% | 73.7% | 0.580 | 35.6% | | 61.9% | 0.218 |

ORR=objective response rate, DCR=disease control rate, CR=complete response, PR=partial response, SD=stable disease, PD=progression disease, PFS=progression free survival, mPFS=median progression free survival, 6m-PFS rate=6 months progression free survival, 12m-PFS rate=12 months progression free survival, OS=overall survival, mOS=median overall survival, m=months, 6m-OS rate=6 months overall survival, 12m-OS rate=12 months overall survival, NR=not reaching, 95% CI=95% confidence interval.

## Table 17 Key efficacy indicators in the inosine and the non-inosine groups of the no lung metastasis and the lung metastasis

|  | **No lung metastasis group** | | | **Lung metastasis group** | | |
| --- | --- | --- | --- | --- | --- | --- |
|  | **Inosine**  (N=70) | **No-inosine**  (N=66) | ***P*** | **Inosine**  (N=16) | **Non-inosine**  (N=20) | ***P*** |
| **ORR**  n (%) | 17  (24.3%) | 12  (18.2%) | 0.385 | 6  (37.5%) | 1  (5.0%) | 0.030 |
| **DCR**  n (%) | 61  (87.1%) | 55  (83.3%) | 0.531 | 15  (93.8%) | 15  (75.0%) | 0.196 |
| CR  n (%) | 0  (0%) | 0  (0%) |  | 0  (0%) | 0  (0%) |  |
| PR  n (%) | 17  (24.3%) | 12  (18.2%) |  | 6  (37.5%) | 1  (5.0%) |  |
| SD  n (%) | 44  (62.9%) | 43  (65.1%) |  | 9  (56.2%) | 14  (70.0%) |  |
| PD  n (%) | 9  (12.9%) | 11  (16.7%) |  | 1  (6.3%) | 5  (25.0%) |  |
| **PFS** |  |  |  |  |  |  |
| events  n (%) | 43  (61.4%) | 58  (87.9%) |  | 11  (68.8%) | 17  (85.0%) |  |
| mPFS (m)  (95% CI) | 7.00  (5.59-8.41) | 4.23  (2.76-5.71) | 0.013 | 5.77  (0.00-12.60) | 4.40  (1.82-6.98) | 0.483 |
| 6-mPFS rate  (%) | 58.1% | 39.5% | 0.040 | 47.1% | 42.5% | 1.000 |
| 12-mPFS rate  (%) | 33.6% | 16.4% | 0.007 | 37.7% | 25.5% | 0.493 |
| **OS** |  |  |  |  |  |  |
| events  n (%) | 17  (24.3%) | 24  (36.4%) |  | 5  (31.3%) | 8  (40.0%) |  |
| mOS (m)  (95% CI) | NR | 29.67  (14.32-45.02) | 0.908 | NR | 24.50  (12.46-36.55) | 0.823 |
| 6-mOS rate  (%) | 88.2% | 92.2% | 0.445 | 75.0% | 89.7% | 0.374 |
| 12-mOS rate  (%) | 72.9% | 70.7% | 0.552 | 66.7% | 72.9% | 0.677 |

ORR=objective response rate, DCR=disease control rate, CR=complete response, PR=partial response, SD=stable disease, PD=progression disease, PFS=progression free survival, mPFS=median progression free survival, 6m-PFS rate=6 months progression free survival, 12m-PFS rate=12 months progression free survival, OS=overall survival, mOS=median overall survival, m=months, 6m-OS rate=6 months overall survival, 12m-OS rate=12 months overall survival, NR=not reaching, 95% CI=95% confidence interval.

## Table 18 Key efficacy indicators in the inosine and the non-inosine groups of the no lymphatic metastasis and the lymphatic metastasis

|  | **No lymphatic metastasis** | | | **Lymphatic metastasis** | | |
| --- | --- | --- | --- | --- | --- | --- |
|  | **Inosine**  (N=17) | **Non-inosine**  (N=13) | ***P*** | **Inosine**  (N=69) | **Non-inosine**  (N=73) | ***P*** |
| **ORR**  n (%) | 3  (17.6%) | 1  (7.7%) | 0.613 | 20(29.0%) | 12  (16.4%) | 0.107 |
| **DCR**  n (%) | 17  (100%) | 11  (84.6%) | 0.179 | 59(85.5%) | 59  (80.8%) | 0.457 |
| CR  n (%) | 0  (0%) | 0  (0%) |  | 0  (0%) | 0  (0%) |  |
| PR  n (%) | 3  (17.6%) | 1  (7.7%) |  | 20  (29.0%) | 12  (16.4%) |  |
| SD  n (%) | 14  (82.4%) | 10  (76.9%) |  | 39  (56.5%) | 47  (64.4%) |  |
| PD  n (%) | 0  (0%) | 2  (15.4%) |  | 10  (14.5%) | 14  (19.2%) |  |
| **PFS** |  |  |  |  |  |  |
| events  n (%) | 10  (58.8%) | 11  (84.6%) |  | 44  (63.8%) | 64  (87.7%) |  |
| mPFS (m)  (95% CI) | 7.10  (5.83-8.37) | 3.43  (2.64-4.23) | 0.112 | 6.93  (5.03-8.84) | 4.43  (2.72-6.15) | 0.027 |
| 6-mPFS rate  (%) | 64.7% | 26.4% | 0.139 | 54.1% | 42.4% | 0.178 |
| 12-mPFS rate (%) | 37.0% | 17.6% | 0.259 | 34.1% | 18.5% | 0.017 |
| **OS** |  |  |  |  |  |  |
| events  n (%) | 3  (17.6%) | 6  (46.2%) |  | 19(27.5%) | 26  (35.6%) |  |
| mOS (m)  (95% CI) | NR | 29.67  (1.40-57.94) | 0.327 | NR | 24.50 | 0.532 |
| 6-mOS rate  (%) | 94.1% | 76.9% | 0.290 | 83.7% | 94.2% | 0.043 |
| 12-mOS rate  (%) | 67.8% | 56.1% | 0.242 | 71.5% | 73.9% | 0.851 |

ORR=objective response rate, DCR=disease control rate, CR=complete response, PR=partial response, SD=stable disease, PD=progression disease, PFS=progression free survival, mPFS=median progression free survival, 6m-PFS rate=6 months progression free survival, 12m-PFS rate=12 months progression free survival, OS=overall survival, mOS=median overall survival, m=months, 6m-OS rate=6 months overall survival, 12m-OS rate=12 months overall survival, NR=not reaching, 95% CI=95% confidence interval.

## Table 19 Key efficacy indicators in the inosine and the non-inosine groups of the non-hepatic metastasis and the hepatic metastasis

|  | **Non-hepatic metastasis** | | | **Hepatic metastasis** | | |
| --- | --- | --- | --- | --- | --- | --- |
|  | **Inosine**  (N=65) | **Non-inosine**  (N=64) | ***P*** | **Inosine**  (N=21) | **Non-inosine**  (N=22) | ***P*** |
| **ORR**  n (%) | 17  (26.2%) | 10  (15.6%) | 0.142 | 6  (28.6%) | 3  (13.6%) | 0.407 |
| **DCR**  n (%) | 59  (90.8%) | 55  (85.9%) | 0.392 | 17  (81.0%) | 15  (68.2%) | 0.337 |
| CR  n (%) | 0  (0%) | 0  (0%) |  | 0  (0%) | 0  (0%) |  |
| PR  n (%) | 17  (26.2%) | 10  (15.6%) |  | 6  (28.6%) | 3  (13.6%) |  |
| SD  n (%) | 42  (64.6%) | 45  (70.3%) |  | 11  (52.4%) | 12  (54.5%) |  |
| PD  n (%) | 6  (9.2%) | 9  (14.0%) |  | 4  (19.0%) | 7  (31.8%) |  |
| **PFS** |  |  |  |  |  |  |
| events  n (%) | 40  (61.5%) | 54  (84.4%) |  | 14  (66.7%) | 21  (95.5%) |  |
| mPFS (m)  (95% CI) | 7.00  (4.78-9.22) | 4.47  (2.46-6.48) | 0.090 | 6.97  (3.67-10.27) | 2.77  (0.20-5.33) | 0.027 |
| 6-mPFS rate  (%) | 55.6% | 43.1% | 0.188 | 57.1% | 31.8% | 0.095 |
| 12-mPFS rate  (%) | 37.7% | 23.7% | 0.054 | 27.5% | 4.5% | 0.042 |
| **OS** |  |  |  |  |  |  |
| events  n (%) | 14  (21.5%) | 21  (32.8%) |  | 8  (38.1%) | 11  (50.0%) |  |
| mOS (m)  (95% CI) | NR | 29.67 | 0.980 | NR | 15.40(14.54-16.27) | 0.714 |
| 6-mOS rate  (%) | 85.8% | 91.9% | 0.284 | 85.4% | 90.5% | 0.956 |
| 12-mOS rate  (%) | 78.2% | 73.7% | 0.487 | 54.2% | 63.8% | 0.666 |

ORR=objective response rate, DCR=disease control rate, CR=complete response, PR=partial response, SD=stable disease, PD=progression disease, PFS=progression free survival, mPFS=median progression free survival, 6m-PFS rate=6 months progression free survival, 12m-PFS rate=12 months progression free survival, OS=overall survival, mOS=median overall survival, m=months, 6m-OS rate=6 months overall survival, 12m-OS rate=12 months overall survival, NR=not reaching, 95% CI=95% confidence interval.

## Table 20 Key efficacy indicators in the inosine and the non-inosine groups of the non-brain metastasis and the brain metastasis

|  | | **Non-brain metastasis** | | | **Brain metastasis** | | |
| --- | --- | --- | --- | --- | --- | --- | --- |
|  | | **Inosine**  (N=81) | **Non-inosine**  (N=73) | ***P*** | **Inosine**  (N=5) | **Non-inosine**  (N=13) | ***P*** |
| **ORR**  n (%) | 22  (27.2%) | | 10  (13.7%) | 0.040 | 1  (20.0%) | 3  (23.1%) | 0.522 |
| **DCR**  n (%) | 71  (87.7%) | | 60  (82.2%) | 0.342 | 5  (100%) | 10  (76.9%) | 1.000 |
| CR  n (%) | 0  (0%) | | 0  (0%) |  | 0  (0%) | 0  (0%) |  |
| PR  n (%) | 22  (27.1%) | | 10  (13.7%) |  | 1  (20.0%) | 3  (23.1%) |  |
| SD  n (%) | 49  (60.5%) | | 50  (68.5%) |  | 4  (80.0%) | 7  (53.8%) |  |
| PD  n (%) | 10  (12.3%) | | 13  (17.8%) |  | 0  (0%) | 3  (23.1%) |  |
| **PFS** |  | |  |  |  |  |  |
| events  n (%) | 52  (64.2%) | | 64  (87.7%) |  | 2  (40.0%) | 11  (84.6%) |  |
| mPFS (m)  (95% CI) | 6.93  (5.66-8.21) | | 4.33  (2.95-5.73) | 0.045 | 13.80 | 4.43  (0.38-8.49) | 0.047 |
| 6-mPFS rate  (%) | 54.5% | | 38.9% | 0.052 | 80.0% | 48.4% | 0.596 |
| 12-mPFS rate  (%) | 31.8% | | 21.2% | 0.034 | 80.0% | 0% | 0.022 |
| **OS** | |  |  |  |  |  |  |
| events  n (%) | | 20  (24.7%) | 24  (32.9%) |  | 2(40.0%) | 8  (61.5%) |  |
| mOS (m)  (95% CI) | | NR | NR | 0.807 | 13.80 | 15.80  (12.21-19.39) | 0.591 |
| 6-mOS rate  (%) | | 11  86.1% | 4  94.3% | 0.090 | 1  80.0% | 3  76.2% | 1.000 |
| 12mOS rate  (%) | | 19  71.6% | 19  71.5% | 0.712 | 1  80.0% | 3  76.2% | 1.000 |

ORR=objective response rate, DCR=disease control rate, CR=complete response, PR=partial response, SD=stable disease, PD=progression disease, PFS=progression free survival, mPFS=median progression free survival, 6m-PFS rate=6 months progression free survival, 12m-PFS rate=12 months progression free survival, OS=overall survival, mOS=median overall survival, m=months, 6m-OS rate=6 months overall survival, 12m-OS rate=12 months overall survival, NR=not reaching, 95% CI=95% confidence interval.

## Table 21 Key efficacy indicators in the inosine and the non-inosine groups of the ≤Ⅲ stage and Ⅳ stage

|  | **≤Ⅲ stage** | | | **Ⅳ stage** | | |
| --- | --- | --- | --- | --- | --- | --- |
|  | **Inosine**  **(N=23)** | **Non-inosine**  **(N=13)** | **P** | **Inosine**  **(N=63)** | **Non-inosine**  **(N=73)** | **P** |
| **ORR**  n (%) | 6  (26.1%) | 2  （15.4%） | 0.682 | 17  (27.0%) | 11  （15.1%） | 0.087 |
| **DCR**  n (%) | 21  (91.3%) | 10  （76.9%） | 0.328 | 55  (87.3%) | 60  （82.2%） | 0.411 |
| CR  n (%) | 0  (0%) | 0  （0%） |  | 0  (0%) | 0  （0%） |  |
| PR  n (%) | 6  (26.1%) | 2  （15.4%） |  | 17  (27.0%) | 11  （15.1%） |  |
| SD  n (%) | 15  (65.2%) | 8  （61.6%） |  | 38  (60.3%) | 49  （67.1%） |  |
| PD  n (%) | 2  (8.7%) | 3  （23.1%） |  | 8  (12.7%) | 13  （17.8%） |  |
| **PFS** |  |  |  |  |  |  |
| events  n (%) | 11  (47.8%) | 10  (76.9%) |  | 43  (68.3%) | 65  (89.0%) |  |
| mPFS (m)  (95% CI) | 8.20  (3.87-12.53) | 4.10  (2.81-5.40) | 0.261 | 6.93  (5.46-8.40) | 4.43  (3.22-5.65) | 0.034 |
| 6-mPFS rate  (%) | 61.1% | 40.9% | 0.458 | 54.5% | 40.1% | 0.111 |
| 12-mPFS rate  (%) | 47.1% | 30.7% | 0.465 | 29.7% | 16.7% | 0.031 |
| **OS** |  |  |  |  |  |  |
| events  n (%) | 1  (4.3%) | 4  (36.4%) |  | 21  (33.3%) | 28  (37.3%) |  |
| mOS (m)  (95% CI) | NR | NR |  | NR | 29.67  (17.64-41.69) |  |
| 6-mOS rate  (%) | 95.7% | 81.8% | 0.239 | 82.3% | 93.1% | 0.049 |
| 12-mOS rate  (%) | 95.7% | 63.6% | 0.029 | 62.7% | 72.5% | 0.527 |

ORR=objective response rate, DCR=disease control rate, CR=complete response, PR=partial response, SD=stable disease, PD=progression disease, PFS=progression free survival, mPFS=median progression free survival, 6m-PFS rate=6 months progression free survival, 12m-PFS rate=12 months progression free survival, OS=overall survival, mOS=median overall survival, m=months, 6m-OS rate=6 months overall survival, 12m-OS rate=12 months overall survival, NR=not reaching, 95% CI=95% confidence interval.

## Table 22 Key efficacy indicators in the inosine and the non-inosine groups of the first line and the second line and above

|  | **First line** | | | **Second line and above** | | |
| --- | --- | --- | --- | --- | --- | --- |
|  | **Inosine**  (N=56) | **Non-inosine**  (N=45) | ***P*** | **Inosine**  (N=30) | **Non-inosine**  (N=41) | ***P*** |
| **ORR**  n (%) | 18  (32.1%) | 9  (20.0%) | 0.171 | 4  (13.3%) | 4  (9.8%) | 0.714 |
| **DCR**  n (%) | 51  (91.1%) | 39  (86.7%) | 0.533 | 24  (80.0%) | 31  (75.6%) | 0.662 |
| CR  n (%) | 0  (0%) | 0  (0%) |  | 0  (0%) | 0  (0%) |  |
| PR  n (%) | 18  (32.1%) | 9  (20.0%) |  | 4  (13.3%) | 4  (9.8%) |  |
| SD  n (%) | 33  (58.9%) | 30  (66.7%) |  | 20  (66.6%) | 27  (65.9%) |  |
| PD  n (%) | 5  (8.9%) | 6  (13.3%) |  | 6  (20%) | 10  (24.4%) |  |
| **PFS** |  |  |  |  |  |  |
| events  n (%) | 37  (66.1%) | 38  (82.6%) |  | 17  (56.6%) | 37  (92.5%) |  |
| mPFS (m)  (95% CI) | 7.10  (5.26-8.94) | 5.77  (2.50-9.03) | 0.161 | 6.93  (3.84-10.02) | 3.50  (2.92-4.08) | 0.094 |
| 6-mPFS rate  (%) | 57.2% | 46.9% | 0.325 | 54.6% | 32.6% | 0.092 |
| 12-mPFS rate  (%) | 35.5% | 22.6% | 0.085 | 31.9% | 13.6% | 0.036 |
| **OS** |  |  |  |  |  |  |
| events  n (%) | 13  (23.2%) | 14  (31.1%) |  | 9  (30.0%) | 18  (43.9%) |  |
| mOS (m)  (95% CI) | NR | 24.50 | 0.900 | 14.10  (9.46-18.74) | 20.67  (7.75-33.59) | 0.336 |
| 6-mOS rate  (%) | 89.2% | 92.9% | 0.761 | 78.3% | 90.1% | 0.315 |
| 12-mOS rate  (%) | 71.0% | 70.1% | 0.681 | 73.0% | 72.8% | 0.954 |

ORR=objective response rate, DCR=disease control rate, PFS=progression free survival, OS=overall survival, mPFS=median progression free survival, mOS=median overall survival, m=months, 6m-PFS rate=6 months progression free survival, 12m-PFS rate=12 months progression free survival, 6m-OS rate=6 months overall survival, 12m-OS rate=12 months overall survival, CR=complete response, PR=partial response, SD=stable disease, PD=progression disease, NR=not reaching, 95%CI=95% confidence interval.
