# Supplementary material for: Inosine enhances the efficacy of immune‐checkpoint inhibitors in advanced solid tumors: A randomized, controlled, Phase 2 study
Source: Cancer Med. 2024 Sep 13;13(17):e70143. doi: 10.1002/cam4.70143 (PMC11393481; doi:10.1002/cam4.70143)
Supplement: Supplementary file 3 — Appendix S3: [file CAM4-13-e70143-s003.docx]

# Appendix 3

**Inosine Enhances the Efficacy of Immune-checkpoint Inhibitors in Advanced Solid Tumors: A Randomized, Controlled, Phase 2 Study**

Haiqing Zhao, Wei Zhang, Yuting Lu, Yin Dong, Zhihao He, Hongchao Zhen, Qin Li*

**Summary**: Inosine had a tendency to enhance the efficacy of ICIs and reduced immunotherapy-related adverse reactions in clinical applications.

## 1 Patient

## Inclusion Criteria：

(1) The interval between the end of palliative care for localized lesions (non-target lesions) and the time of randomization was required to be longer than 3 weeks.

(2) Please provide archived or fresh pathological tissues that have been tested for programmed death-ligand.1(PD-L1), Mismatch repair gene (MMR)/Microsatellite instability (MSI), Tumor mutation burden (TMB) within 6 months from the date of signing the screening informed consent form.

(3) Adequate organ and bone marrow function is defined as follows: 1) blood routine: an absolute neutrophil count ≥1.5×10^9/L, a platelet count ≥100×10^9/L, and a hemoglobin level ≥9.0g/dL. 2) Liver function: patients without liver metastases are required to have a serum total bilirubin (TBIL) level ≤1.5×upper limits of normal (ULN), alanine aminotransferase (ALT) and aspartate aminotransferase (AST) levels ≤2.5 × ULN. Patients with liver metastases are required to have TBIL levels ≤1.5×ULN; ALT and AST levels ≤5×ULN. 3) Renal function: creatinine clearance rate (Ccr) ≥50 mL/min (calculated by the Cockcroft/Gault formula): for females, Ccr = (140 - age) × weight (kg) × 0.85; for males, Ccr = (140 - age) × weight (kg) × 1.00). 4) Adequate coagulation function is defined as having an international normalized ratio (INR) or prothrombin time (PT) ≤1.5 × ULN.

(4) Expected survival time ≥ 12 weeks

(5) Female subjects of childbearing age or male subjects whose sexual partner is a female of childbearing age are required to use effective contraception throughout the treatment period and for 6 months thereafter

## Exclusion Criteria：

(1) History of intestinal obstruction or the following diseases: inflammatory bowel disease or extensive bowel resection, Crohn's disease, ulcerative colitis.

(2) Hepatic metastatic burden of approximately 50% or more of total liver volume.

(3) Antibiotic was used within 2 weeks before study treatment.

(4) Symptomatic congestive heart failure (New York Heart Association class≥3) or symptomatic or poorly controlled arrhythmias.

(5) Patients used immunosuppressive medications, excluding topical glucocorticoids administered through nasal spray, inhalation, or other routes, as well as the use of physiologic doses of systemic glucocorticoids (i.e., no more than 10 mg/day of prednisone or an equivalent dose of other glucocorticoids), and the use of hormones for preventing contrast allergy within 4 weeks prior to study treatment

(6) Patients had interstitial lung disease and hormone therapy.

(7) Patients had Active, known or suspected autoimmune disease or history of such disease within the previous 2 years (patients with vitiligo, psoriasis, alopecia areata, or Grave's disease who do not require systemic treatment within the last 2 years, hypothyroidism requiring only thyroid hormone replacement therapy, and type I diabetes requiring only insulin replacement therapy may be enrolled)

(8) Uncontrolled hypertension (systolic blood pressure ≥ 160 mmHg or diastolic blood pressure ≥ 100 mmHg) even with standardized treatment

(9) Any arterial thromboembolic event, including myocardial infarction, unstable angina, cerebrovascular accident, or transient ischemic attack, within 6 months prior to enrollment in treatment

(10) History of deep vein thrombosis, pulmonary embolism, or any other serious thromboembolism within 3 months prior to enrollment

(11) Have a history of active tuberculosis

(12) Have a history of human immunodeficiency virus infection

(13) Hepatitis B surface antigen-positive and peripheral blood hepatitis B virus deoxyribonucleic acid (HBV-DNA) titer test ≥ 1 × 104 copies/mL (or HBV-DNA quantification is ≥ 2000 units/ml); active hepatitis C.

(14) History of any other primary malignancies, except for malignancies that have been in complete remission for at least 2 years prior to enrollment and do not require further treatment during the study; adequately treated non-melanoma skin cancers or malignant freckle-like nevus with no evidence of disease recurrence; adequately treated carcinoma evidence of disease recurrence.

(15) Other patients who, in the opinion of the investigator, may interfere with the conduct of the clinical trial, may not be able to comply with the protocol, may not be able to cooperate, or may pose a risk to the trial.
